# Amyloid β toxic conformer has dynamic localization in the human inferior parietal cortex in absence of amyloid plaques

**DOI:** 10.1038/s41598-018-35004-3

**Published:** 2018-11-15

**Authors:** Yusuke Kageyama, Atsushi Saito, Olga Pletnikova, Gay L. Rudow, Yumi Irie, Yang An, Kazuma Murakami, Kazuhiro Irie, Susan M. Resnick, David R. Fowler, Lee J. Martin, Juan C. Troncoso

**Affiliations:** 1 Department of Pathology, The Johns Hopkins University School of Medicine, Baltimore, MD 21205 USA; 2 Department of Neuroscience, The Johns Hopkins University School of Medicine, Baltimore, MD 21205 USA; 3 Department of Neurology, The Johns Hopkins University School of Medicine, Baltimore, MD 21205 USA; 4 Division of Food Science & Biotechnology, Graduate School of Agriculture, Kyoto University, Kyoto, Japan; 5 Laboratory of Behavioral Neuroscience, NIH/NIA/IRP, Baltimore, MD USA; 6 Office of the Chief Medical Examiner, Baltimore, MD USA; 7 Present Address: Shiga University of Medical Science, Otsu, Shiga, 520-2192 Japan

## Abstract

Amyloid β (Aβ) plays a critical role in the pathogenesis of Alzheimer’s disease. Nevertheless, its distribution and clearance before Aβ plaque formation needs to be elucidated. Using an optimized immunofluorescent staining method, we examined the distribution of Aβ in the post-mortem parietal cortex of 35 subjects, 30 to 65 years of age, *APOE* ε3/ε3, without AD lesions. We used 11A1, an antibody against an Aβ conformer which forms neurotoxic oligomers. 11A1 immunoreactivity (IR) was present in cortical neurons, pericapillary spaces, astrocytes and the extracellular compartment at 30 years of age. The percentage of neurons with 11A1 IR did not change with age, but the number and percentage of astrocytes with 11A1 IR gradually increased. Notably, the percentage of pericapillary spaces labeled with 11A1 IR declined significantly in the 5^th^ decade of the life, at the same time that 11A1 IR increased in the extracellular space. Our findings indicate that the Aβ toxic conformer is normally present in various cell types and brain parenchyma, and appears to be constitutively produced, degraded, and cleared from the inferior parietal cortex. The decrease in pericapillary Aβ and the concomitant increase of extracellular Aβ may reflect an age-associated impairment in Aβ clearance from the brain.

## Introduction

Alzheimer’s disease (AD) is the most common cause of dementia in old age. Its pathological hallmarks are deposition of amyloid beta (Aβ) plaques and tau-based neurofibrillary tangles in the cerebral cortex associated with inflammatory changes, and degeneration of neurons and synapses^1,2^. Autopsy and neuroimaging studies (Aβ positron emission tomography [PET]) have demonstrated that the deposition of Aβ in the brain precedes the onset of cognitive decline by a decade or more^3–6^. However, the biology of Aβ before the emergence of plaques in the human brain is still poorly understood.

It is known that Aβ can adopt several oligomeric forms and their increased tissue concentration precedes the formation of Aβ plaques^7,8^. A study of amyloid precursor protein (APP) metabolism *in vivo* and vitro reported enormous and rapid production of Aβ in neurons and its release into the extracellular compartment^9^. The constant metabolism of APP and gradual increase of Aβ oligomers prior to Aβ plaque formation implicate an efficient clearance system of Aβ oligomers in the brain. The mechanisms for clearance of extracellular Aβ from the brain are multiple^10^ and include local enzymatic degradation^11^, transport across the blood brain barrier (BBB)^12–15^, via the glymphatic system^16–18^ and CSF^19^. The relative contributions of these various systems to Aβ clearance are not fully understood.

In order to understand the pathological mechanisms that lead to the accumulation of Aβ and the formation of plaques in the brain in AD, it is critical to first understand the physiological clearance of this peptide in young subjects free of classical Aβ lesions. To this end, it is necessary to characterize the presence of Aβ oligomers in various brain compartments at the cellular and subcellular levels prior to plaque formation. The identification of the precise cellular and subcellular localizations of Aβ will help to understand the mechanism of Aβ clearance before plaque formation. This information can be used to generate hypotheses relevant to the sequential steps in the clearance of Aβ, the modification or impairment of this clearance in aging, and can open new avenues for therapeutic intervention.

In this study, we used the 11A1 monoclonal antibody to monitor the localization of an Aβ species with a specific molecular structure designated as Aβ toxic conformer. As revealed by solid-state NMR assessment, Aβ adopts at least two conformations; one with a turn at positions 22 and 23, and the other with a turn at positions 25 and 26^20^. The particular Aβ conformation with a turn at positions 22 and 23 brings the Tyrosine at position 10 (Tyr-10) and the Methionine at position 35 (Met-35) close together and causes formation of an S-oxidized radical cation of Met-35^21^. This redox reaction stabilizes this Aβ conformation which shows increased aggregation leading to Aβ oligomer formation and neurotoxicity, and is designated Aβ toxic conformer^21,22^. Formation of Aβ toxic conformer is facilitated in Aβ42 compared to Aβ40 due to the physical distance between Tyr-10 and Met-35, which is longer in Aβ40^23^. The 11A1 antibody is designed to target the specific structure of the Aβ toxic conformer^24^. Previous studies have established that the major target of the 11A1 antibody is the Aβ42 toxic conformer including its oligomeric forms. However, weak immunoreactivity against Aβ40 toxic conformer and Aβ42 monomer cannot be excluded. Thus, hereafter, we use the term “11A1 immunoreactivity” (11A1 IR) to encompass all of these Aβ targets. This antibody has demonstrated intracellular and extracellular Aβ in brains of AD patients^24,25^, mouse models of AD^25,26^, and neurons derived from IPSCs from AD patients^27^.

Here, we characterized the localization of Aβ toxic conformer using 11A1 antibody. To this end, we examined autopsy tissues of the inferior parietal cortex in subjects 30 to 65 years of age, found histologically free of Aβ plaques and tau pathology, and all of them with *apolipoprotein E (APOE)* ε3/ε3 genotype. We observed that approximately 85% of cortical neurons, 75% of protoplasmic astrocytes and 30% of pericapillary spaces showed 11A1 IR at 30 years of age. Furthermore, we also observed extracellular 11A1IR, in the form of particles not associated with neuronal and astrocytic cell bodies or pericapillary spaces. The 11A1 labeling of neurons remained stable throughout the age spectrum and that of astrocytes showed a gradual increase. Notably, during the 5^th^ decade of the life the number of pericapillary spaces labeled with 11A1 IR began to decline as the extracellular labeling began to increase. These concomitant changes suggest a possible impairment in the pericapillary clearance of Aβ from the inferior parietal cortex becoming evident in the 5^th^ decade of the life.

## Results

### Optimized immunofluorescence procedure on human brain with tissue quality screening

**Figure 1:**
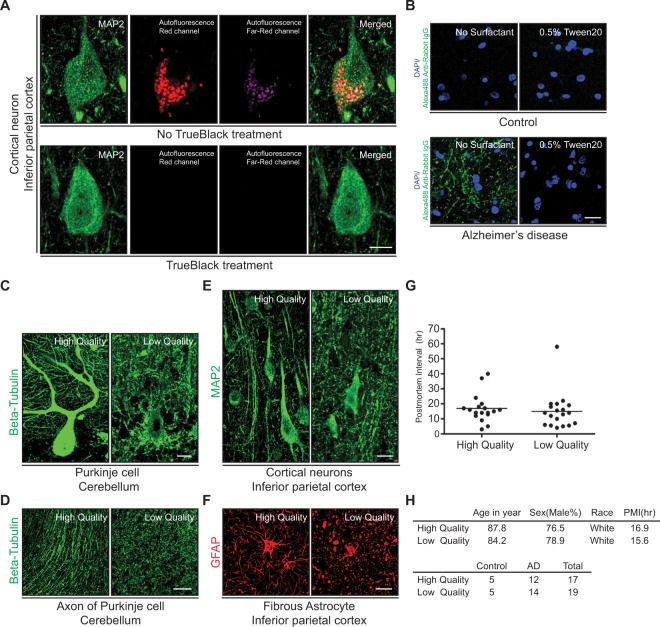
Optimized immunofluorescence procedure on human brain with tissue quality screening. (**A**) Quenching of lipofuscin autofluorescence signal in human brain sections with TrueBlack treatment. Confocal imaging of inferior parietal layer V pyramidal cortical neurons immunostained with MAP2 antibody are shown on the far left panels. The two central panels illustrate the autofluorescence of lipofuscin in the red and far-red channels. The far right panels show the merged images. The top panel illustrates results in the absence of TrueBlack treatment and shows prominent lipofuscin autofluorescence in the red and far red channels and in the merged image. The bottom panel shows complete quenching of the lipofuscin signal with TrueBlack treatment. The images were taken under 63x objective lens. *Scale bars* 5 µm. (**B**) Quenching of nonspecific binding of anti-rabbit IgG secondary antibody. Tissues from brains with AD changes had marked non-specific binding of anti-rabbit IgG which can be prevented by 0.5% Tween20 in secondary antibody incubation buffer. The top panel shows section of parietal cortex from a control subject. The bottom panel corresponds to parietal cortex from an AD case. On the left, in the absence of surfactant there is prominent non-specific binding of anti-rabbit IgG, which disappears in the presence of 0.5% Tween20. The images were taken under 20x objective lens. *Scale bars* 20 µm. (**C** and **D**) Purkinje cells of the cerebellum immunostained with Beta-tubulin antibody. In (**C**), the left panel shows the structural integrity of the cell body and dendrites in a high-quality tissue sample. In contrast, the right panel shows the loss of immunostaining and disintegration of dendrites in a low-quality tissue sample. The images were taken under 20x objective lens. *Scale bars* 20 µm. In (**D**), the left panel shows well preserved axonal morphology and the right panel shows fragmentation of axons. The images were taken under 10x objective lens. *Scale bars* 50 µm. (**E**) Neurons of the parietal cortex immunostained with MAP2 antibody. The left panel shows well preserved cell bodies and dendrites. The right panel demonstrates fragmentation of dendrites and deformed cell bodies. The images were taken under 20x objective lens. *Scale bars* 20 µm. (**F**) Parietal cortex white matter astrocytes immunostained for GFAP. Left panel shows intact cell bodies and processes, which are disintegrating in the right panel. The images were taken under 20x objective lens. *Scale bars* 20 µm. (**G**) Tissue quality versus postmortem interval. Postmortem tissues (n = 36) were classified as high- or low-quality based on the integrity of Purkinje cells as described in (**C**). The plots show that there is no correlation between postmortem interval and tissue preservation (p = 0.5907). The bars in the graph represents the mean of postmortem interval in each group. (**H**) Demography and neuropathological diagnoses of autopsied subjects assessed in (**G**).

In order to examine the localization of Aβ oligomers in the cells, interstitial space, and blood vessels of the human brain, we used immunohistochemistry with fluorescent secondary antibodies combined with confocal microscopy. This approach, however, has two major obstacles that hinder the interpretation of fluorescent labeled images: prominent autofluorescent signal from neuronal lipofuscin and non-specific binding of anti-rabbit IgG secondary antibody^28,29^. Thus, we set forth to establish a reliable staining method on postmortem formalin-fixed paraffin-embedded human brain tissue that can circumvent the aforementioned hurdles. We found that a treatment of antibody labeled tissue with TrueBlack^TM^ Lipofuscin Autofluorescence Quencher with a modification of the manufacturer’s protocol successfully quenches autofluorescence signals without dimming the signal intensity from fluorescent conjugated antibodies (Fig. 1A, Supplementary Fig. S1). Next, we searched for a method to quench non-specific binding of anti-rabbit secondary antibody on human brain tissue. Tissues from age-matched control subjects free of neuropathological changes did not show significant non-specific binding of the antibody (Fig. 1B). In contrast, tissues from AD patients displayed prominent non-specific binding of the secondary antibody (Fig. 1B, Supplementary Table S2). This non-specific binding was successfully quenched by using 0.5% Tween20/PBS for the incubation of the secondary antibody as previously described^29^. Only anti-rabbit IgG secondary antibody exhibited this non-specific binding, whereas anti-mouse, anti-guinea pig and anti-chicken IgG secondary antibodies did not show a similar pattern. Interestingly, the non-specific binding of the secondary antibody was noted not only in tissues from AD patients, but also in tissues from subjects with various neurodegenerative disorders (Supplementary Fig. S2, Supplementary Table S1). In all examined cases, non-specific binding of the antibody was quenched with 0.5% Tween20/PBS (Supplementary Fig. S2).

Next, we aimed for a method to gauge tissue preservation and to ascertain that these autopsy cases can be successfully used for our study. To this end, we decided to examine directly neuronal populations particularly susceptible to hypoxic-ischemic injury. It is well established that pyramidal neurons in the CA1 region of the hippocampus and Purkinje cells in the cerebellum, are particularly vulnerable to hypoxic-ischemic injury in the human brain^30^. Since Purkinje cells in the cerebellum are spared in the majority of neurodegenerative disorders, we reasoned that the morphological status of these neurons would provide an index of brain tissue preservation independent from neurodegenerative changes. In some brains, Purkinje cells immunostained with beta-tubulin showed intact cell bodies, unbroken and smooth dendrites, and delicate bundles of axons with preserved varicosities (Figs. 1C, 1D, Supplementary Fig. S3). Tissues from these brains were designated as “high-quality samples”. In other brains, Purkinje neurons showed disintegrating cell bodies, dendrites, and axons (Fig. 1C,D, Supplementary Fig. S3). We designated tissues from these brains as “low-quality samples”. When we examined the morphology of neurons and astrocytes in various regions of the brain of the high-quality samples, intact neuronal and astrocytic cell bodies and their unbroken dendrites and processes were observed in all examined regions (Fig. 1E,F, Supplementary Fig. S4). In contrast, disintegrating dendrites and astrocytic processes were prominently noted in various regions of the brains of low-quality samples (Fig. 1E,F, Supplementary Fig. S4). Therefore, we concluded that the morphology of Purkinje neurons is a reliable indicator of overall postmortem brain tissue preservation.

In many quarters, the postmortem interval (PMI) is a well-accepted criterion for the assessment of the preservation of brain tissue. We examined the relation between PMI and structural integrity of Purkinje cells in autopsy cases with accurate PMI documented from the Baltimore Longitudinal Study of Aging (BLSA). Notably, we could not find a correlation between PMI and structural integrity of Purkinje cells (Fig. 1G). The demography and neuropathology of examined subjects showed no marked difference (Fig. 1H). This data suggests a substantial impact for pre- and perimortem circumstances on the preservation of autopsy tissues. Previous studies revealed that not only PMI, but premortem factors, such as agonal state, course of disease progression and tissue pH shift due to antemortem acidosis, significantly influence tissue preservation^31,32^. Tissue quality assessed based on the structural integrity of Purkinje cells is consistent with previous studies, and appears as a practical and direct approach to assess tissue preservation independently of pre- and postmortem factors.

### Assessment of 11A1 affinity and immunoreactivity

**Figure 2:**
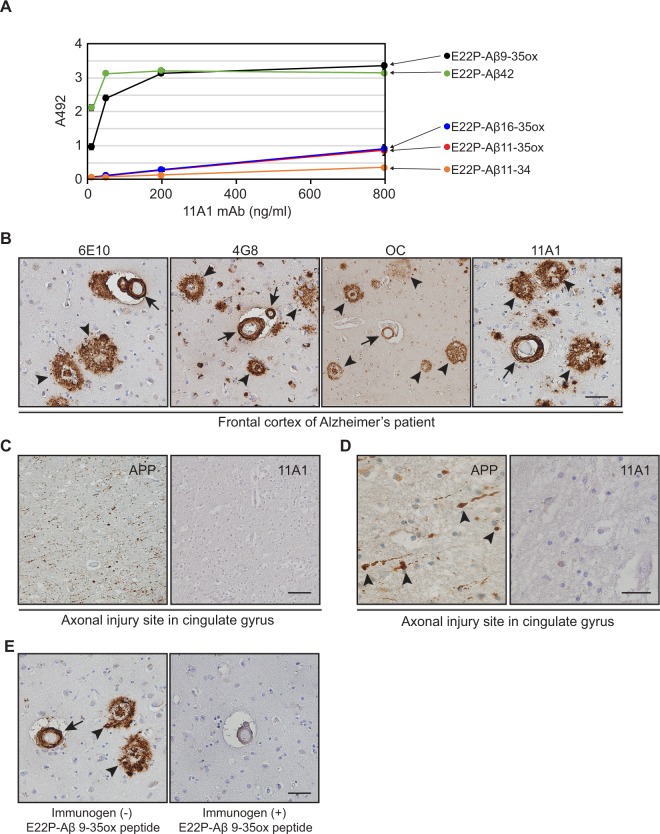
Characterization of immunoreactivity of 11A1 antibody. (**A**) Assessment of the binding affinity of 11A1 antibody against various synthetic Aβ peptides. E22P-Aβ42 (green), E22P-Aβ9-35ox (black), E22P-Aβ11-35ox (red), E22P-Aβ16-35ox (blue), and E22P-Aβ11-34 (orange) were synthesized and the binding affinity of 11A1 antibody for each peptide was assessed by Enzyme immunoassay. The 11A1 antibody exhibited high binding affinity for E22P-Aβ42 and E22P-Aβ9-35ox. Data are expressed as mean ± SD of three independent measurements. (**B**) Comparison of 11A1 antibody staining pattern with that of 6E10, 4G8, and OC antibodies. Postmortem human frontal cortex sections of an Alzheimer’s disease patient were stained with 6E10 (left panel), 4G8 (center left panel), OC (center right panel) and 11A1 (right panel) antibodies. Senile plaques (arrow heads) and vascular amyloid (arrows) are identified by 11A1 antibody with a similar pattern to that of the other three Aβ antibodies. The images were taken under 20x objective lens. *Scale bar* 50 µm. (**C** and **D)** Assessment of 11A1 cross-reactivity against APP. Histological sections of human cingulate gyrus with traumatic axonal injury were stained with 22C11 (APP antibody) (left panel) and 11A1 (right panel) antibodies. The traumatic axonal balloons display intense signal with the antibody for APP (arrowheads), but these lesions are not stained with 11A1 antibody. (**C**) The images were taken under 20x objective lens. *Scale bar* 50 µm. (**D**) The images were taken under 40x objective lens. *Scale bar* 20 µm. (**E**) Testing 11A1 antibody specificity for Aβ toxic conformer in senile plaques and blood vessels. 11A1 antibody was pre-incubated without (left panel) or with (right panel) its immunogen, E22P-Aβ9-35ox peptide, prior to incubation of tissues with the primary antibody. The pre-incubation with immunogen abrogates the signal in senile plaques and blood vessels. The images were taken under 20x objective lens. *Scale bar* 50 µm.

First, specificity of the 11A1 antibody against the target structure of Aβ was assessed with various synthetic Aβ peptides with EIA. Amino acid at position 22 and 23 of synthetic Aβ peptides are modified to Pro-X corner (E22P) to stabilize the turn structure^24^. 11A1 antibody showed high binding affinity against E22P-Aβ42 and E22P-Aβ9-35ox peptides (Fig. 2A). In contrast, the 11A1 antibody showed weak immunoreactivity against E22P-Aβ11-34, E22P-Aβ11-35ox and E22P-Aβ16-35ox (Fig. 2A). E22P-Aβ11-34 is missing Tyr-10 and Met-35, E22P-Aβ11-35ox and E22P-Aβ16-35ox are missing Tyr-10. These data demonstrate the specificity of 11A1 for the Aβ structure with a turn at positions 22 and 23 and stabilized with a redox reaction of Tyr-10 and Met-35.

Next, we optimized the conditions for immunohistochemistry with the 11A1 antibody on human brain tissue, specifically pretreatment with formic acid and antigen retrieval with EDTA. 11A1 IR against senile plaques was highest with formic acid/EDTA antigen retrieval treatment, less intense with formic acid alone, barely detectable with antigen retrieval alone, and absent with no treatment (Supplementary Fig. S5). Next, we used the same four conditions to test the signal intensity and specificity of the 11A1 antibody against intracellular Aβ in the inferior parietal cortex of a normal subject. Immunoreactivities of 11A1 and MAP2 antibodies were robust and comparable with formic acid/EDTA antigen retrieval and EDTA antigen retrieval, but absent in the other two conditions (Supplementary Fig. S6). From these results, formic acid/EDTA antigen retrieval treatment appears as the optimal condition for 11A1 staining of Aβ plaques and intracellular Aβ. We confirmed the same immunostaining pattern of 11A1 with that of other conventional Aβ antibodies: 6E10 4G8 and OC^33^. All antibodies recognized senile plaques and vascular Aβ on frontal cortex sections of an AD patient that were pre-treated with formic acid/EDTA antigen retrieval (Fig. 2B). 11A1 cross-reactivity against APP was assessed on histological sections of human cingulate gyrus with traumatic axonal injury and prominent axonal balloons which are enriched in APP^34^. Whereas, these axonal balloons were strongly immunostained by the anti-APP antibody 22C11, the 11A1 showed no immunoreactivity (Fig. 2C,D). This result indicates no cross-reactivity of 11A1 antibody against APP on human brain tissue immunohistochemistry confirming the biochemical observation of Murakami *et al*.^24^. To further confirm the specificity of 11A1 antibody against Aβ toxic conformer, abrogation of staining by the immunogen of 11A1 antibody was performed. Pre-incubation of the 11A1 antibody with its immunogen, E22P-Aβ9-35 (CYEVHHQKLVFFAPDVGSNKGAIIGLM) in 200-molar excess for 1 hour at 37 °C, abrogated the 11A1 staining from senile plaques and blood vessels (Fig. 2E).

We confirmed that brain tissues treated with formic acid/EDTA antigen retrieval show clear staining patterns of Aβ plaques and intracellular Aβ with 11A1 antibody. However, a question remained as to whether these staining conditions are compatible with other antibodies used in this study for co-staining with 11A1, especially due to harshness of formic acid treatment and potential damage to the antigen structure. We compared the immunoreactivity of other antibodies used in this study with formic acid/EDTA antigen retrieval, EDTA antigen retrieval or no treatment. The immunoreactivity of all antibodies, except CD9, was enhanced by EDTA antigen retrieval treatment (Supplementary Fig. S6, middle and right panels). Immunoreactivity of all antibodies was indistinguishable when comparing formic acid/EDTA antigen retrieval versus EDTA antigen retrieval treatment (Supplementary Fig. S6, left and middle panels).

### 11A1 IR in pericapillary spaces

**Figure 3:**
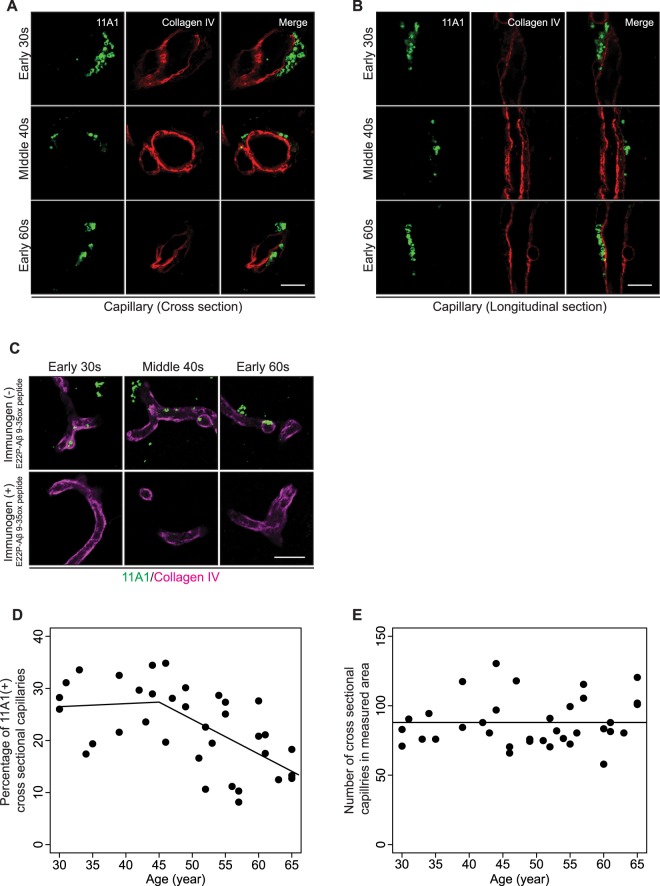
11A1 immunoreactivity in pericapillary spaces. (**A** and **B**) Representative images were obtained under 63x objective lens. Pericapillary spaces in inferior parietal cortex, layer V, immunostained with 11A1 and Collagen IV. The rows of figures correspond to three age groups: early 30 s, middle 40 s, and early 60 s. Left panels show the 11A1 signal, middle panels indicate the Collagen IV signal and right panels show merged images. (**A**) Capillary cross sections, (**B**) capillary longitudinal sections. *Scale bars* (**A** and **B)**, 5 µm. (**C**) Verification of 11A1 antibody’s specificity against Aβ toxic conformer in pericapillary space of the three age groups. The 11A1 antibody was pre-incubated with (bottom panels) or without (top panels) its immunogen, E22P-Aβ9-35ox peptide, prior to incubation of tissues with the primary antibody. The pre-incubation with immunogen abrogates the 11A1 signal around Collagen IV signal (magenta). Images were obtained under 20x objective lens. *Scale bar* 20 µm. (**D**) Percentage of pericapillary spaces labeled with 11A1 in measured area across age-spectrum (30-65 years). The relationship with age is not statistically significant before age 45 (p = 0.82), but it becomes highly significant after age 45 (p = 0.0006) by the Piecewise regression. (**E**) Number of cross sectional pericapillary spaces in measured area across age-spectrum (30–65 years). There is no significant change in the number of cross sectional capillary pericapillary spaces with age (*P* = 0.496).

First, we discovered the presence of 11A1 IR in pericapillary spaces identified with Collagen IV and Glial fibrillary acidic protein (GFAP) co-staining. (Movies S1, S2). Both in longitudinal and cross-sectional profiles, the 11A1 signal (green) was associated with spherical/vesicular particles 1–2 μm in size, sandwiched between the GFAP signal of astrocytes (magenta) and the Collagen IV signal of the pericapillary matrix (red). 11A1 immunoreactive particles were present in the pericapillary spaces of 30-year-old subjects and their pattern of staining showed no marked difference with age (Fig. 3A,B). Pre-incubation of the antibody with its immunogen abolished the 11A1 signals observed in the pericapillary spaces (Fig. 3C). Interestingly, the number of 11A1 IR-labeled pericapillary spaces appears to remain stable until the 5th decade of life, when they start to decrease both in longitudinal and cross-sectional profiles (Supplementary Fig. S7). We measured the fraction of 11A1 labeled cross-sectional pericapillary spaces as described in Methods. Regression analyses comparing intercept only, linear with age, and piecewise with age (knot at age 45 years) models showed that the piecewise regression model fitted the best (using Akaike information criterion [AIC], Supplementary Table S2), indicating that this relationship is not linear. Approximately 30% of cross-sectional pericapillary spaces showed an 11A1 signal between ages 30 and 45 years and its association with age was not statistically significant (p = 0.818). But this percentage significantly declined after 45 years of age (p = 0.0006, Fig. 3D). For the total number of the cross-sectional pericapillary spaces in the measured area, the intercept only model fitted the data best, meaning that this parameter did not have a significant association with age (p = 0.483, Fig. 3E).

### 11A1 IR in neurons

**Figure 4:**
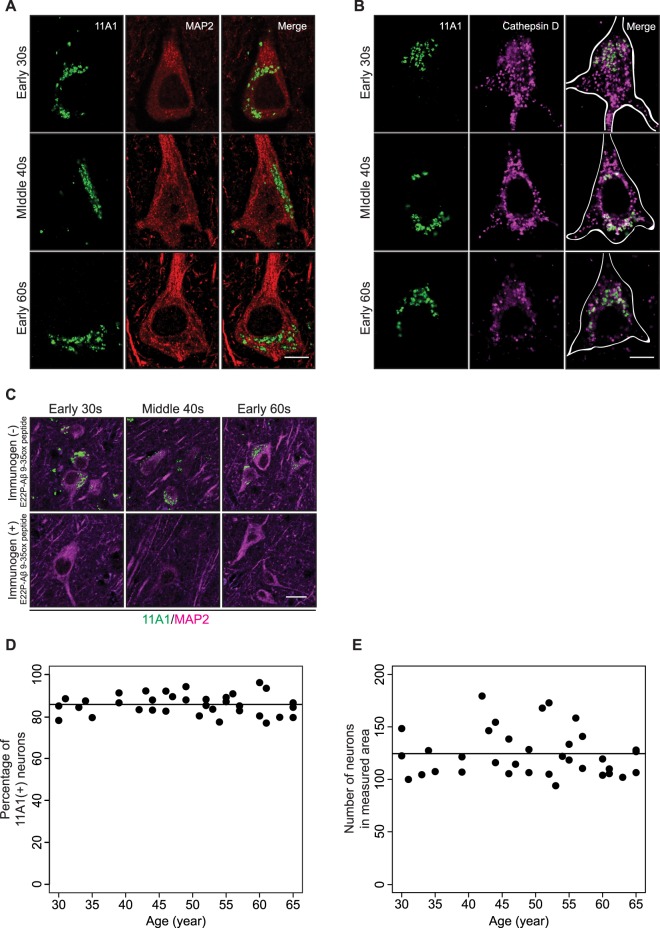
11A1 immunoreactivity in neurons. (**A** and **B**) Representative images were obtained under 63x objective lens. Cortical neurons of inferior parietal cortex, layer V, co-stained with 11A1 and MAP2 antibodies and 11A1, MAP2 and Cathepsin D antibodies. The rows of figures correspond to three age groups: early 30 s, middle 40 s, and early 60 s. (**A**) Left panels show the 11A1 signal (green), middle panels indicate MAP2 signal (red) and right panels show merged images. *Scale bar* 5 µm. (**B**) Left panels show the 11A1 signal, middle panels indicate Cathepsin D signal and right panels show merged images. The white contours in the merged images indicates the cell shape identified by MAP2. Intracellular 11A1 immunoreactivity (green) partially colocalized with Cathepsin D signal (Magenta). *Scale bar* 5 µm. (**C**) Verification of 11A1 antibody’s specificity against Aβ toxic conformer in neurons of the three age groups. 11A1 antibody was pre-incubated with (bottom panels) or without (top panels) its immunogen, E22P-Aβ9-35ox peptide, prior to incubation of tissue sections with the primary antibody. The pre-incubation with immunogen abrogates the 11A1 signal (green) in and around MAP2 signals (magenta). Images were obtained under 20x objective lens. *Scale bar* 20 µm. (**D**) Percentage of cortical neurons labeled with 11A1 in measured area across the age-spectrum (30–65 years). The relationship with age is not significant (p = 0.807). (**E**) Number of neurons in measured area across the age-spectrum (30–65 years). There is no significant change in the number of neurons with age (p = 0.6061).

Second, we discovered that inferior parietal neurons characterized by MAP2 immunoreactivity displayed 11A1 signal in their perikaryon (Fig. 4A). Notably, 11A1 IR was already present in the cortical neuron of 30-year-old subjects and its pattern of staining showed no marked change with age. The subcellular localization of 11A1 IR was further tested with co-incubation of the 11A1 antibody with a battery of markers for cellular organelles: Cathepsin D (lysosome), Rab5 (early endosome), LC3 (autophagosome), p62 (autophagosome). We found that Cathepsin D partially colocalized with 11A1 signals in the neurons across the age spectrum (Fig. 4B). These data suggest degradation of intraneuronal Aβ in the lysosomal compartment. Pre-incubation of the antibody with its immunogen abolished the 11A1 signals observed in the neuron (Fig. 4C), confirming that the 11A1 signal in neurons is related to the Aβ toxic conformer. Lastly, we measured the fraction of 11A1 labeled cortical neurons as described in Methods. Regression analyses showed the intercept model fitted the data best (Supplementary Table S2), which means that this percentage and the total number of neurons in the measured area did not show significant relationships with age. (p = 0.807 and 0.595 respectively Fig. 4D,E). Approximately 85% of cortical neurons showed the 11A1 signal across the age spectrum.

### 11A1 IR in microglia

Third, Microglia activation is one of pathological hallmarks of AD^35,36^. We examined whether 11A1 immunoreactive particles colocalize with microglia in young subjects. The majority of microglia were free of 11A1 IR in subjects 30 to 65 years of age (Supplementary Fig. S8A–C). As previously reported, we also confirmed that microglia is present in the Aβ plaques of an AD case (Supplementary Fig. S8D). These data suggest that microglia activation is a late and reactive event after the formation of Aβ plaques.

### 11A1 IR in protoplasmic astrocytes

**Figure 5:**
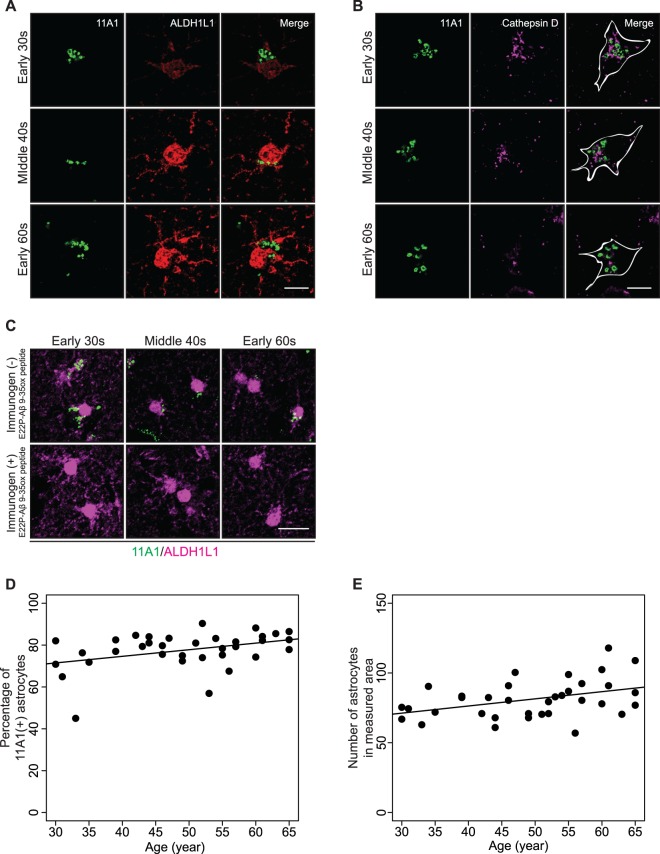
11A1 immunoreactivity in protoplasmic astrocytes. (**A** and **B**) Representative images were obtained under 63x objective lens. Protoplasmic astrocytes of human inferior parietal cortex, layer V, immunostained with 11A1 and ALDH1L1 antibodies and 11A1, GFAP and Cathepsin D antibodies. The rows of figures correspond to three age groups: early 30 s, middle 40 s, and early 60 s. **(A)** Left panels show the 11A1 signal (green), middle panels correspond to the ALDH1L1 signal (red) and right panels show the merged images. *Scale bar* 5 µm. (**B**) Left panels show the 11A1 signal, middle panels correspond to the Cathepsin D signal (magenta) and right panels show the merged images. The white contours in the merged images indicate the cell shape identified by GFAP. Separate localization of the 11A1 immunoreactivity and Cathepsin D signals in the astrocytes is demonstrated. *Scale bar* 5 µm. (**C**) Verification of 11A1 antibody’s specificity against Aβ toxic conformer in protoplasmic astrocytes of the three age groups. The 11A1 antibody was pre-incubated with (bottom panels) or without (top panels) its immunogen, E22P-Aβ9-35ox peptide, prior to incubation of tissue sections with primary antibody. The pre-incubation with immunogen abrogates the 11A1 signal (green) in and around ALDH1L1 signal (magenta). Images were obtained under 20x objective lens. *Scale bar* 20 µm. (**D**) Percentage of protoplasmic astrocytes labeled with 11A1 of the astrocytes in measured area across the age-spectrum (30–65 years). The percentage of 11A1 labeled astrocytes gradually increases by age (p = 0.020). (**E**) Number of the astrocytes in measured area across the age-spectrum (30–65 years). Number of astrocytes gradually increases by age (p = 0.014).

Fourth, we observed the presence of 11A1 IR in protoplasmic astrocytes co-staining with 11A1 and ALDH1L1 (Fig. 5A). 11A1 IR was present in astrocytes of 30-year-old subjects, and the pattern of staining showed no marked difference with age. Subcellular localization of 11A1 IR was further tested with co-incubation of 11A1 antibody with the Cathepsin D and various degradation markers. Cathepsin D, p62, Ubiquitin, Rab5 and 11A1 signals in protoplasmic astrocytes did not show a marked colocalization pattern (Fig. 5B, Supplementary Fig. S9). These data suggest different Aβ processing mechanisms in neurons and astrocytes. Pre-incubation of the antibody with its immunogen abolished the 11A1 signal observed in astrocytes (Fig. 5C). Lastly, we measured the percentage of 11A1 labeled protoplasmic astrocytes as described in Methods. Regression models with linear age fitted the data best (Supplementary Table S2). Approximately 75% of the astrocytes showed the 11A1 signal at age 30 years, and both the percentage of labeled astrocytes and total number of astrocytes exhibited a slight yet significant increase with age. (p = 0.020 and 0.014 respectively Fig. 5D,E).

### Extracellular 11A1 IR

**Figure 6:**
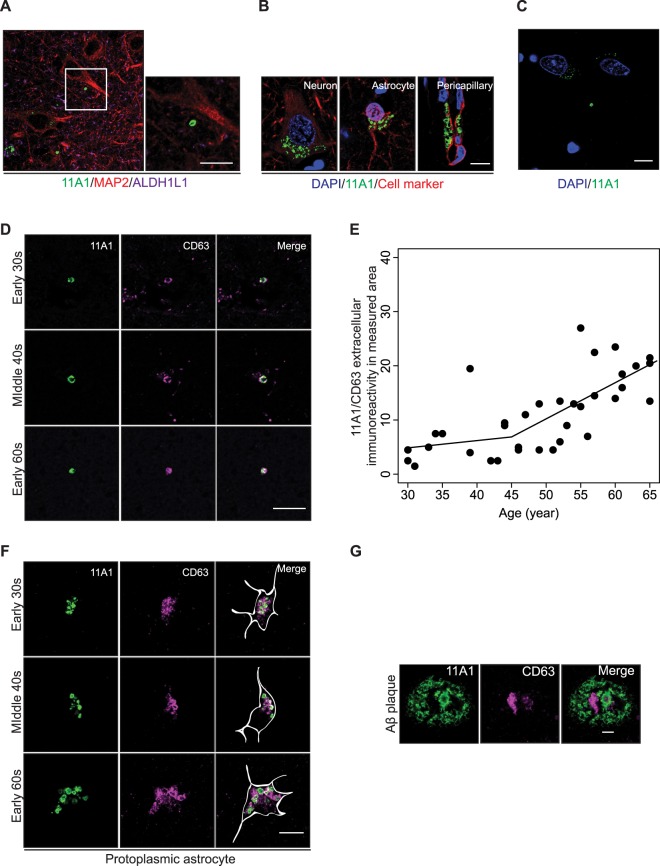
Extracellular 11A1 immunoreactivity. (**A**) Representative images of an extracellular 11A1 immunoreactivity of inferior parietal cortex, layer V (63x objective lens). Left panel shows the merged image of 11A1 (green), MAP2 (red) and ALDH1L1 (magenta) signals. The area outlined by the white rectangle is magnified in the right panel and shows the extracellular 11A1 immunoreactivity. *Scale bar* 5 µm. (**B**) Representative immunostaining patterns of 11A1 antibody in neurons, astrocytes and pericapillary spaces in human cerebral cortex (63x objective lens). The row of figures shows immunostaining with 11A1 (green) and cellular/compartment marker (red) antibodies combined with DAPI staining. The cellular marker for neuron is MAP2 and for astrocyte is ALDH1L1, for pericapillary space is Collagen IV. The 11A1 immunoreactive particles form clusters around the DAPI signal in neurons and astrocytes. In the pericapillary space, there are both clusters and rows of 11A1 immunoreactive particles near the DAPI signal (blue). *Scale bar* 5 µm. (**C**) Staining pattern of the extracellular 11A1 immunoreactivity (green) ≥5 µm apart from DAPI signals (blue). In this compartment, the 11A1 immunoreactive particles do not form clusters, as seen in Fig. 6B, but appear as single and isolated structures independent of neuronal and astrocytic cell bodies and of blood vessels. *Scale bar* 5 µm. (**D**) Colocalization of the extracellular 11A1 immunoreactivity (green) with CD63 signal (magenta). The criteria for the identification of the extracellular 11A1 immunoreactivity is described in the method section. The rows of figures correspond to three age groups: early 30 s, middle 40 s, and early 60 s. *Scale bar* 5 µm. (**E**) Total number of the extracellular 11A1 immunoreactivity in measured area across the age-spectrum (30–65 years). The relationship with age is not statistically significant before age 45 (*P* = 0.51), but it becomes highly significant after age 45 (*P* < 0.0001) by the Piecewise regression. (**F**) Colocalization of 11A1 (green) and CD63 (magenta) immunoreactivity in protoplasmic astrocytes. The rows of figures correspond to three age groups: early 30 s, middle 40 s, and early 60 s. Left panels show the 11A1 signal, middle panels the CD63 signal, and right panels the merged images. The white line in the merged images indicates the cell shape identified by GFAP signal. *Scale bar* 5 µm. (**G**) Representative image of 11A1 labeled Aβ plaque and CD63 signal. Left panels show the 11A1 labeled Aβ plaque (green), middle panels the CD63 signal (magenta), and right panels the merged images. *Scale bar* 5 µm.

Lastly, we observed 11A1 IR which did not colocalize with neuronal or astrocytic bodies or pericapillary spaces and termed it as “extracellular 11A1 IR” (Fig. 6A). The 11A1 signal in neurons, astrocytes and pericapillary spaces appeared as clusters in close proximity to DAPI labeled cellular nuclei (Fig. 6B). In contrast, the extracellular 11A1 IR appears as single particles with <1 µm in diameter, distant ≥5 µm from the edge of DAPI signal (Fig. 6C). We set as criterion for identification of extracellular 11A1 immunoreactive particles a distance ≥5 µm from DAPI signals. From their hollow shape, we speculated that extracellular 11A1particles could be related to vesicles and performed colocalization analyses with vesicle markers: CD9, CD63, Flotillin1, TSG101^37,38^. None of these markers showed colocalization with the extracellular 11A1 immunoreactive particle (Supplementary Fig. S10), except for CD63 (Fig. 6D). We measured the number of extracellular 11A1 immunoreactive particles co-staining with CD63 as described in Methods. Piecewise regression model fitted best for its relationship with age (Supplementary Table S2). The number of extracellular 11A1/CD63 particles per area remained stable until age 45 years (p = 0.506) and increased sharply thereafter (p < 0.0001) (Fig. 6E).

Next, we tested the expression of CD63 in various cell types to determine the origin of these particles. CD63 was expressed in protoplasmic astrocytes, and the 11A1 IR in the astrocytes colocalized with the CD63 signal (Fig. 6F). These data suggest that the extracellular 11A1 particles may be processed in astrocytes, which also express CD63 (Fig. 6F). We also tested whether other vesicular markers such as CD9, TSG101, and Flotillin in neurons and astrocytes colocalize with 11A1 particles. However, they did not colocalize well with 11A1 IR. (Supplementary Fig. S11). Furthermore, we observed localization of the CD63 signal in the 11A1 labeled Aβ plaques of three individuals at sixth decades of life, all noted with sparse Aβ plaques in the cerebral cortex. In concert, these observations suggest that the extracellular 11A1/CD63 particles may have a role in the early stages of Aβ plaques formation (Fig. 6G).

## Discussion

The aim of this study was to examine the localization of the Aβ toxic conformer identified with 11A1 antibody in the human inferior parietal cortex before the development of Aβ plaques or deposits in the parenchyma or blood vessels. Our observations show that 11A1 IR is present in neurons, in astrocytes, in pericapillary spaces and in the extracellular compartment but is not substantially associated with microglia. The association of 11A1 IR with neurons remains stable throughout the examined age span (30–65 years) and the number and percentage of 11A1 labeled astrocytes increases gradually and slightly with age. In contrast, we observed that approximately at age 45 years, the percentage of 11A1-labeled pericapillary spaces decreases sharply. This decline of 11A1-labeled pericapillary spaces suggests a possible impairment of Aβ clearance from the inferior parietal cortex. Since pericapillary spaces are connected with the cerebrospinal fluid (CSF), our findings are consistent with reported lower levels of Aβ in the CSF of older individuals, and MCI or AD patients^39,40^ and with PET scan observations in living subjects indicating that Aβ starts to accumulate in the brain of normal subjects about age 50 years^41^. Furthermore, our finding of increased extracellular 11A1 IR is consistent with these observations and at the same time supports the notion that the decreased number of 11A1–labeled pericapillary spaces contributes to a decline in Aβ clearance from the brain in the fifth decade of life.

Since the Aβ plaques of AD are extracellular aggregates, the majority of studies of Aβ have focused on the extracellular compartment of the brain rather than the intraneuronal compartment. Nevertheless, a substantial body of evidence has developed pointing towards the presence of Aβ in the neurons^42–44^, and not only in AD^45,46^ and Down syndrome^44,47^, but also in control subjects^48,49^. The distribution of 11A1 signal in the perikarya of cortical neurons had a punctate pattern suggestive of localization within organelles. Of several markers tested, we observed colocalization of the 11A1 IR with Cathepsin D. This observation is consistent with the processing of Aβ by the lysosomal system^50,51^. Both the 11A1 and Cathepsin D labelings were comparable across the age range. Furthermore, we found that both the number of neurons and the proportion of 11A1-labeled neurons in cortical layer V remained stable across the age spectrum. Taking these data together, we speculate that under physiological conditions, neurons maintain a steady level of intracellular Aβ with a lysosomal degradation system. Further studies identifying the precise localization of intraneuronal Aβ at the early stages of AD, might shed light on the potential role of intraneuronal Aβ on the pathological mechanism of plaque formation.

Previous studies have reported Aβ incorporation into astrocytes in the aged human brain^52–54^. Here we found that this event occurs at an early stage of life, since approximately 75% of protoplasmic astrocytes are labeled with 11A1, and this proportion remains unchanged from age 30 to 65 years. Notably, Aβ particles in the astrocytes do not strongly colocalize with Cathepsin D as observed in neurons, but with CD63, a tetraspanin associated with exosomes and extracellular microvesicles^38^. Extracellular 11A1 IR and Aβ plaques also showed CD63 signal, implicating a possible involvement of astrocytes in formation of extracellular 11A1 IR and Aβ plaques. Although astrocytes express an order of magnitude less APP than neurons (http://www.brainrnaseq.org/)^55^, we cannot exclude the possibility that astrocytes may also generate Aβ. We also observed that astrocytes exhibited a slight yet significant increase with age. It has been estimated that in the brain of rodents, the number of astrocytes and pericytes increases 20% in the cortex and other brain regions in older animals^56–59^.

Stereological studies in humans, however, have shown no increase in the number of cortical astrocytes in aging in subjects ages 18 to 93 years^60^ or 65 to 105 years^61^. The difference in our observations on the number of astrocytes with previous studies could be explained by our focus on a very limited region (inferior parietal) or by differences in quantitative techniques. It is of interest that we did not observe Aβ oligomers in the microglia, which is commonly present in AD brains^62^. This can attributed to the absence of insoluble Aβ or Aβ deposits in the tissues we examined, whereas in AD brains Aβ deposits are abundant, in plaques and blood vessels, and elicit the activation and phagocytic activity of microglia^63^. Previous studies indicate that impairment of Aβ clearance via perivascular spaces from the brain may be associated with AD^17,64^. In this study, individual or clusters of 11A1 immunoreactive particles were noted in pericapillary spaces between the GFAP signal of astrocytic endfeet and the signal of Collagen IV, one of the constituents of the perivascular matrix^16^. The presence of 11A1 immunoreactive particles in the pericapillary space suggests that this is a clearance pathway for the particles. Notably, we found that the fraction of pericapillary spaces with 11A1 immunoreactive particles declined in the 5^th^ decade of life (Fig. 3D). Our study has limitations intrinsic to the examination of human autopsies such as the deterioration of tissue integrity. We tried to circumvent this limitation by a rigorous selection of tissues with demonstrable integrity of cerebellar Purkinje cells. Another limitation is the lack of clinical or cognitive information on the subjects whose brains we examined. For this reason, we are not making any clinical assertions and limiting our conclusions to morphological parameters. A third caveat is that the examination of extracellular particles was limited to confocal microscopy and that we did not employ electron microscopy (EM) for a better characterization of these particles because the fixation of the tissues were not appropriate for EM. Future studies contemplate EM characterization of the extracellular particles.

An important caveat to our study is that our observations of Aβ toxic conformer were conducted in the brains of subjects who did not have Aβ deposits or plaques, therefore we cannot predict whether they would have developed AD or not had they lived longer. Since AD is different from normal aging^65^, the extrapolation of changes in Aβ clearance in normal individuals compared to patients with AD remains speculative. Future studies on the brains of subjects with ApoE4 alleles could be informative in this regard because it is known that this allele enhances the risk for development of AD^66^.

In conclusion, our observations reveal the possibility of perturbations in the pericapillary clearance of the Aβ toxic conformer from the human inferior parietal cortex with aging. The Aβ toxic conformer appears to be in equilibrium in various brain compartments until 5^th^ decade of life. The disruption of this equilibrium may account for aging being the major risk factor for the development of Aβ deposits in the brain and for the eventual development of AD. Our study has the strength of providing novel information on the fate of Aβ in the inferior parietal cortex before the onset of pathologic changes, and of raising new testable hypotheses that may be relevant to the pathogenesis and prevention for the treatment of AD.

## Methods

### Autopsy and Neuropathological examination

Autopsies were performed at the Office of The Chief Medical Examiner (OCME) of the State of Maryland in Baltimore and brains were accessioned by the Lieber Institute for Brain Development (LIBD) for a brain repository program following protocols authorized by the Institutional Review Board (IRB) of the State of Maryland Department of Health and Human Services. The methods were in accordance with the guidelines as determined by the Johns Hopkins Medicine-Institutional Review Board. The Johns Hopkins Medicine-Institutional Review Board has determined the use of unidentified human autopsy tissue is exempt under 45 CFR 46.101 (b) (4), dated April 1, 2003, Application NO:03-03-13-03e, Activities of the Neuropathology Core C (Alzheimer’s Disease). Following external examination and weighing, brains were cut in coronal slabs, diagnostic tissue blocks dissected, and the remainder tissues were frozen. For microscopic examination, tissues from the superior frontal gyrus, middle and superior temporal gyri, inferior parietal cortex, occipital cortex, amygdala, hippocampus and entorhinal cortex, midbrain, pons, medulla, and cerebellum were obtained in all brains. Tissues were fixed in 10% buffered formaldehyde, processed, embedded in paraffin, and cut at 10 µm thickness. All sections were stained with H&E and screened for AD lesions using silver stains (Hirano method)^67^, and immunostained for Aβ (6E10) and tau (PHF-1). From a cohort of 431 autopsy brains from subjects 30–65 years of age, we selected brains free of Aβ plaques and tau pathology in the inferior parietal cortex, then chose those with *APOE* ε3/ε3 and high-quality tissue preservation as described below. At the end of this multistage selection process, we identified 35 brains suitable for the present study. All immunostainings were conducted in tissue sections from the inferior parietal cortex. We chose this region based on our previous neuropathological observations in this cohort^68^ which demonstrated that the early deposition of Aβ is more common in the neocortex, including the inferior parietal cortex, than in the hippocampus. Also, the inferior parietal cortex is one of the cortical regions examined for the standardized neuropathologic assessment of AD according to CERAD guidelines^69^. The demography and causes of death of subjects examined in this study are listed in Table 1. We also examined tissues from AD cases and controls from the Baltimore Longitudinal Study of Aging (BLSA)^70,71^ to examine the impact of postmortem interval on tissue preservation assessed by structural integrity of Purkinje cells of the cerebellum.

**Table 1:** Demography and cause of death of subjects.

Sample#	Sex	Age	Race	Cause of Death
1	M	30	African American	Pneumonia
2	M	30	Caucasian	Heart disease
3	M	31	African American	Diabetic ketoacidosis
4	M	33	Caucasian	Cardiovascular disease
5	F	34	Caucasian	Pneumonia
6	F	35	Caucasian	Heart disease
7	M	39	African American	Pulmonary thromboembolism
8	F	39	Caucasian	Cardiovascular disease
9	F	42	Other	Cardiac arrest
10	M	43	Caucasian	Cardiovascular disease
11	F	44	African American	Cardiovascular disease
12	M	44	Caucasian	Cardiovascular disease
13	M	46	Caucasian	Cardiovascular disease
14	M	46	Caucasian	Myocardial infarct
15	M	47	Caucasian	Cardiovascular disease
16	M	49	Caucasian	Cardiovascular disease
17	M	49	Caucasian	Cardiovascular disease
18	M	51	Caucasian	Cardiovascular disease
19	M	52	African American	Cardiovascular disease
20	F	52	Caucasian	Cardiac arrest
21	M	53	Caucasian	Injuries from motor vehicle accident
22	M	54	Caucasian	Cardiovascular disease
23	M	55	Caucasian	Cardiovascular disease
24	M	55	Caucasian	Heart disease
25	M	56	Caucasian	Cardiovascular disease
26	F	57	Caucasian	Unknown, history of anorexia nervosa
27	M	56	Caucasian	Cardiovascular disease
28	F	60	Caucasian	Overdose of prescription drug
29	F	60	Caucasian	Injuries from motor vehicle accident
30	F	61	Caucasian	Overdose of prescription drug
31	F	61	African American	Heart disease
32	M	63	Caucasian	Heart disease
33	F	65	African American	Injuries from motor vehicle accident
34	M	65	Caucasian	Cardiovascular disease
35	M	65	Caucasian	Heart disease

### Genotyping

We extracted DNA in 413 subjects in which frozen tissue was available and genotyped for *APOE* using the method of Hixon and Vernier^72^.

### Optimized immunofluorescence procedure on human brain with tissue quality screening

This protocol consists of an optimized immunofluorescence procedure for formalin fixed paraffin embedded brain tissue, which includes a preliminary step for ascertaining the optimal preservation of the postmortem tissues. For immunofluorescence staining, sections were deparaffinized in xylene and rehydrated in 100% and 95% EtOH. The samples to be co-stained with the 11A1 antibody were incubated in 70% formic acid (Sigma: F0507) for 12 min at room temperature (RT) prior to antigen retrieval. After washing with tap water, antigen retrieval was performed in 1 mM EDTA (pH8.0) (Invitrogen: 15575-038) by boiling for 4 min. For samples not co-stained with 11A1 monoclonal antibody, antigen retrieval was performed in 1x Citrate buffer (pH6.0) (Abcam: ab93678) by boiling for 4 min. Then, all samples were blocked in PBS (Phosphate Buffered Saline) with 5% normal goat serum and 0.2% Triton x-100 for 1 h at RT. Primary antibodies were incubated in blocking buffer for 16 h at 4 °C. On the following day, samples were washed in PBS for 5 min x 3, then Alexa fluor secondary antibodies in PBS with 0.5% Tween 20 were applied and incubated for 1 h at RT. Samples were washed in PBS once for 5 min, then 5 µg/mL Hoechst 33258 in PBS were applied and incubated for 20 min at RT. Samples were washed in PBS once for 5 min. For quenching lipofuscin autofluorescence, we used TrueBlack^TM^ Lipofuscin Autofluorescence Quencher (Biotium: 23007) diluted 1/40 in 70% EtOH, applied to the samples and incubated for 50 s at RT. To facilitate the TrueBlack reaction, samples were constantly swirled by hand during the incubation. Then, samples were washed in PBS for 5 min x 3 and coverslipped using ProLong^TM^ Gold Antifade reagent (Invitrogen: P36930). Samples were kept at 4 °C at least 2 days before imaging. Immunofluorescent images were taken on a Zeiss LSM 700 confocal microscope in the Microscope Facility of the Johns Hopkins School of Medicine.

For an assessment of brain tissue preservation, we examined the morphology of Purkinje cells of the cerebellum immunostained with a beta-tubulin antibody (Abcam: ab18207). Three criteria are set to assess the morphology of Purkinje cells: cell body shape, dendritic morphology and the features of axonal bundles. The high-quality samples exhibit intact cell bodies, unbroken and smooth dendrites, and delicate bundles of axons with preserved varicosities. Any sample not satisfying all three criteria is considered of low-quality. A 5 × 5 panel montage image using a 10x objective lens is taken of the folia of the cerebellum and every Purkinje cell in the panel encompassing an area of 9.983mm^2^ has to satisfy the high-quality criteria. (Supplementary Fig. S3)

### Immunohistochemistry with 3,3′-Diaminobenzidine (DAB) development

Sections were deparaffinized in xylene and rehydrated in 100% and 95% EtOH. The tissues were incubated in 3% hydrogen peroxidase solution for 15 min at RT. The samples to be stained with 11A1, 6E10 4G8 and OC^33^ antibodies were incubated in 70% formic acid (Sigma: F0507) for 12 min at RT prior to antigen retrieval. After washing with tap water, antigen retrieval was performed in 1 mM EDTA (pH8.0) (Invitrogen: 15575-038) by boiling for 4 min. An exception to this protocol was the sample stained with APP antibody (22C11), which was not treated with 70% formic acid, and antigen retrieval was performed in Citrate buffer (pH6.0) by boiling for 4 min. Then, samples were blocked in PBS with 5% normal goat serum and 0.2% Triton x-100 for 1 h at RT. Primary antibodies were incubated in blocking buffer for 16 h at 4 °C. On the following day, samples were washed in PBS for 5 min x 3, then biotinylated anti-mouse IgG (H + L) (vector: BA-2000) in PBS with 0.5% Tween 20 was applied and incubated for 1 hour at RT. Samples were washed in PBS once for 5 min x 3, and the ABC reagent prepared as instructed by the manufacturer was applied and incubated for 1 hour at RT. Samples were washed in PBS once for 5 min x 3, and DAB (Vector: SK-4100) solution was applied and incubated for 3 min at RT as instructed by manufacturer. Color developed samples were counterstained with hematoxylin (Thermo Scientific: 7211). After dehydration and serial incubation in xylene, samples were coverslipped. Images were taken on an Olympus BX51TF with a DP70 color camera in the Microscope Facility of the Johns Hopkins School of Medicine.

### Characterization of the 11A1 antibody

First, specificity of the 11A1 antibody against the target structure of Aβ was assessed with various synthetic Aβ peptides (described below) by Enzyme immunoassay (EIA) (described below). Second, we optimized the conditions for immunohistochemistry with the 11A1 antibody on human brain tissues. We tested four different conditions of tissue treatment: formic acid/EDTA antigen retrieval treatment, formic acid alone, EDTA antigen retrieval alone, or no treatment. We compared the signal intensity and specificity of 11A1 antibody using frontal cortex sections of an AD patient and the inferior parietal cortex of a normal subject. Third, we compared the staining pattern of the 11A1 antibody with that of other conventional Aβ antibodies, i.e., 6E10 and 4G8 and OC on the frontal cortex tissue sections of an AD patient with formic acid/EDTA antigen retrieval pre-treatment. Fourth, we assessed for 11A1 cross-reactivity against APP on histological sections of the human cingulate gyrus with traumatic axonal injury.

### Synthesis of the fragment peptides of E22P-Aβ42

The fragment peptides of E22P-Aβ42 for ElA of 11A1 were synthesized in a stepwise fashion on 0.1 mmol of preloaded Fmoc-L-Met-PEG-PS or Fmoc-L-Leu-PEG-PS resin by a synthesizer, Biotage^**®**^ initiator + Alstra^TM^ (Biotage Japan) using the Fmoc method. The synthesis of E22P-Aβ9-35(ox) was reported previously^24^. Each coupling reaction was carried out using each Fmoc amino acid (0.4 mmol), HATU (0.4 mmol), and DIPEA (0.8 mmol) in 2.4 mL of DMF for 5 min at 75 °C (10 min at 50 °C for the coupling of Fmoc-L-His) under microwave irradiation. After each coupling reaction, the *N*-terminal Fmoc group was deblocked with 20% piperidine in DMF. After the completion of chain elongation followed by Fmoc deblocking, each peptide resin, washed with DMF and CH_2_Cl_2_, was shaken for 2 h at RT in a mixture containing TFA, *m*-cresol, ethanedithiol, and thioanisole for final deprotection and cleavage from the resin. Each crude peptide was precipitated by diethylether, followed by purification using HPLC on an X-Bridge column (Waters Corporation, USA) and elution at 8.0 mL/min by a 60-min linear gradient (curve 6) of 15–40% CH_3_CN containing 0.1% NH^4^OH for E22P-Aβ11-35(ox) and E22P-Aβ16-35(ox), and by an 80-min exponential gradient (curve 7) of 15–60% CH_3_CN containing 0.1% NH_4_OH for E22P-Aβ11-34. Lyophilization gave each pure peptide, the purity of which was confirmed by LC-qTOF-MS (>95%). The yield and molecular weight of each E22P-Aβ42 fragment peptides were as follows: E22P-Aβ11-35(ox), 7.4% yield, m/z 2723.61 (calcd for [M (Av.)] 2724.20); E22P-Aβ16-35(ox), 5.6% yield, m/z 2094.09 (calcd for [M (Av.)] 2093.53); E22P-Aβ11-34, 11.9% yield, m/z 2577.18 (calcd for [M (Av.)] 2577.00).

### Enzyme immunoassay (EIA)

In a 96-well Maxisorp plate (Nunc), each Aβ fragment peptide (500pmol/well) dissolved in 50 mM sodium carbonate was incubated for 2 h at RT, followed by blocking with 5% bovine serum albumin at 4 °C overnight, as previously described^24^. Briefly, after incubation of 11A1 antibody (IBL) with each peptide clone^24^ for 1 h at RT, the plate was treated with a horseradish peroxidase-coupled anti-mouse IgG antibody (IBL), and quantified using *o*-phenylenediamine dihydrochloride substrate (Sigma) before measurements at 492 nm with a microplate reader (MultiScan JX; Thermo Scientific).

### Antibodies

The information of primary and secondary antibodies used in this study is listed in Supplementary Tables S3 and S4.

### Imaging and data analysis

All images were captured from layer V of the inferior parietal cortex unless otherwise specified in the results section. The cortical layer V was identified as the lamina of pyramidal neurons closest to the gray-white matter junction (Fig. S1). To calculate the percentage of 11A1 labeled cortical neurons and protoplasmic astrocytes, first we immunostained tissue with 11A1, MAP2 and ALDH1L1 antibodies and captured two montage images, one at the crown of the gyrus, and the other midway between the crown of the gyrus and the bottom of the sulcus. Each montage consisted of a 3 × 3 panel of images captured with a 20x objective lens encompassing an area of 0.910mm^2^. The number of neurons and astrocytes with and without 11A1 signals in the measured field were counted to calculate the fraction of the cells with 11A1 signal. We stained an adjacent tissue section with 11A1 and Collagen IV antibodies to calculate the percentage of 11A1 labeled pericapillary spaces using the same imaging approach employed for neurons and astrocytes. For construction of the 3D animation of the pericapillary space with Aβ toxic conformer, first we immunostained the tissue with 11A1, GFAP and Collagen IV antibodies and captured Z-stack images with confocal microscopy. Z-stack images were captured at a setting of 1024 × 1024 pixels, speed 7, on a single scan and at a 0.37 µm interval. The Z-stack images were reconstructed in 3D animation and converted to movies using Bitplane Imaris Microscopy Image Analysis Software. For the quantification of pericapillary spaces labeled with 11A1 signals, we selected only cross-sectional pericapillary spaces with diameters ≤20 µm to avoid interpretation problems arising from measuring longitudinal profiles. The number of pericapillary spaces with and without 11A1 signal in the measured field were counted and used to calculate the fraction of 11A1 labeled pericapillary spaces. The calculated value from the two montage images in each sample was averaged for the final analysis. For the quantification of extracellular 11A1 IR particles co-labeled with CD63, first we immunostained the tissue with the 11A1 and CD63 antibodies followed with DAPI incubation. Then, we captured two montage images of the cortical layer V as described above. The setting of these montages consisted of a 4 × 4 panel of images captured with a 40x objective lens encompassing an area of 0.407 mm^2^ (Supplementary Fig. S12). The number of 11A1 signals co-staining with the CD63 signal was measured in the area. The calculated value from two montage images in each sample was averaged for the final analysis.

### Statistical analyses

The Student’s *t* test was used to compare mean postmortem interval between lower-quality and high-quality of tissue preservation (Fig. 1G). Pearson correlations were used to quantify the linear relationship between age and distribution of IR. To further characterize the relationship of parameters with age, we compared 3 regression models with increasing complexities. The first model is the intercept only model, which means there is no age relationship. The second model is the intercept and age model, which assumes linear age relationship. The third model is the piecewise regression model with knot at age 45 years, which assumes that age relationships are different before and after age 45 years. The models were compared using Akaike information criterion (AIC) (Supplementary Table S2). Statistical analyses were performed with Graphpad Prism 4 or R version 3.4.4.

## Electronic supplementary material

Supplementary Movie 1

Supplementary Movie 2

Supplementary Information

## Data Availability

The authors confirm that the data supporting the findings of this study are available within the article and its supplementary materials.
