# Supplementary material for: Amyloid β toxic conformer has dynamic localization in the human inferior parietal cortex in absence of amyloid plaques
Source: Sci Rep. 2018 Nov 15;8:16895. doi: 10.1038/s41598-018-35004-3 (PMC6237870; doi:10.1038/s41598-018-35004-3)
Supplement: Supplementary file 3 — Supplementary Information [file 41598_2018_35004_MOESM3_ESM.pdf]

Amyloid β toxic conformer has dynamic localization in the human inferior parietal cortex in absence of amyloid plaques

Yusuke Kageyama[1#], Atsushi Saito[1], Olga Pletnikova[1], Gay L. Rudow[1], Yumi Irie[4], Yang An[5], Kazuma Murakami[4], Kazuhiro Irie[4], Susan M. Resnick[5], David R. Fowler[6], Lee J. Martin[1,2], & Juan C. Troncoso[1,3*]

*Correspondence to Juan C. Troncoso (troncoso@jhmi.edu)
Phone: +1-410-955-5632
Fax: +1-410-955-9777

Affiliations: Department of Pathology[1], Department of Neuroscience[2], Department of Neurology[3], The Johns Hopkins University School of Medicine, Baltimore, MD 21205, USA
Division of Food Science & Biotechnology[4], Graduate School of Agriculture, Kyoto University, Kyoto, Japan
Laboratory of Behavioral Neuroscience[5], NIH/NIA/IRP, Baltimore, MD, USA
Office of the Chief Medical Examiner[6], Baltimore, MD, USA

#Current address: Shiga University of Medical Science, Otsu, Shiga, 520-2192, Japan

Supplementary Fig .S1

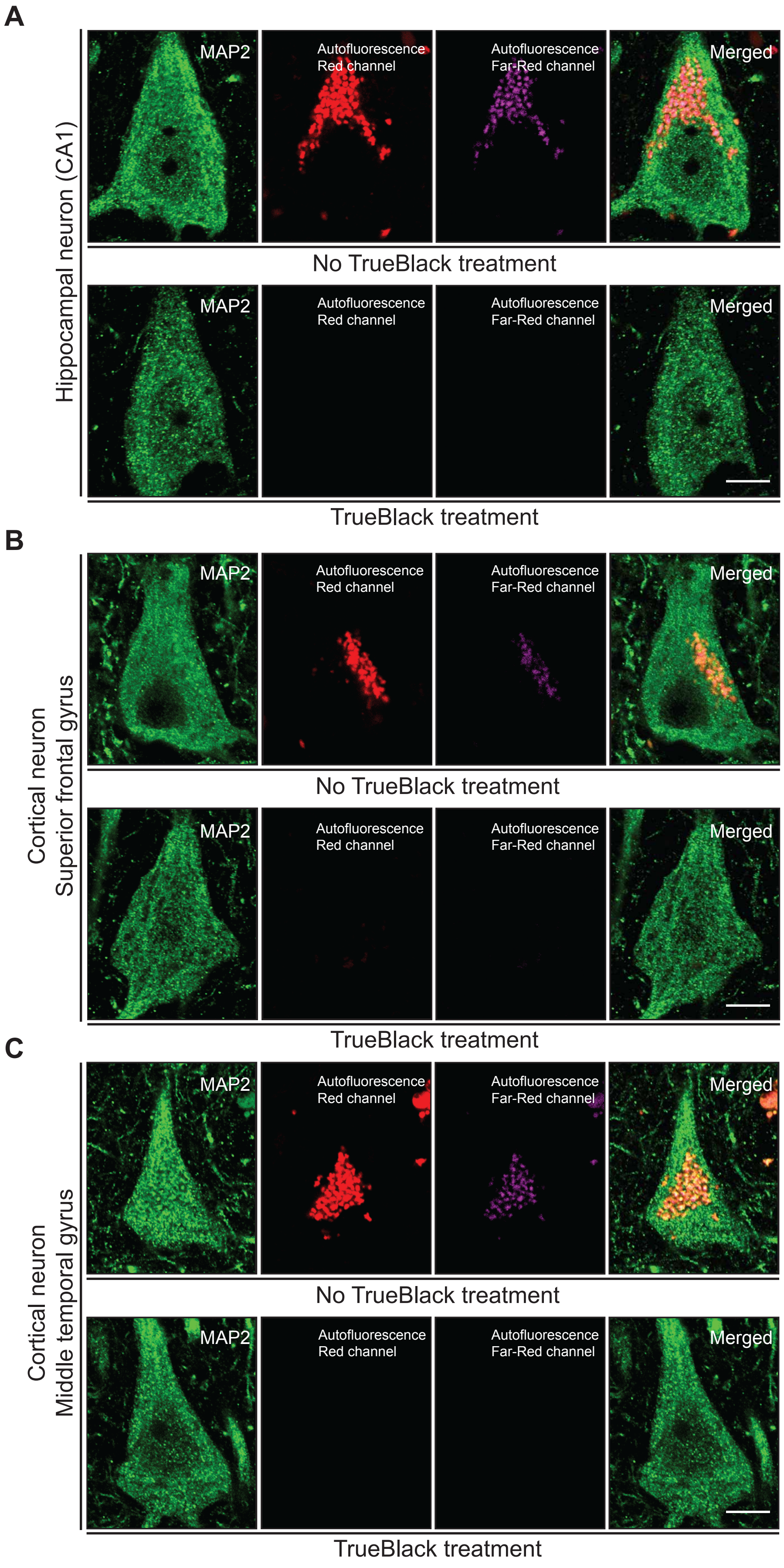

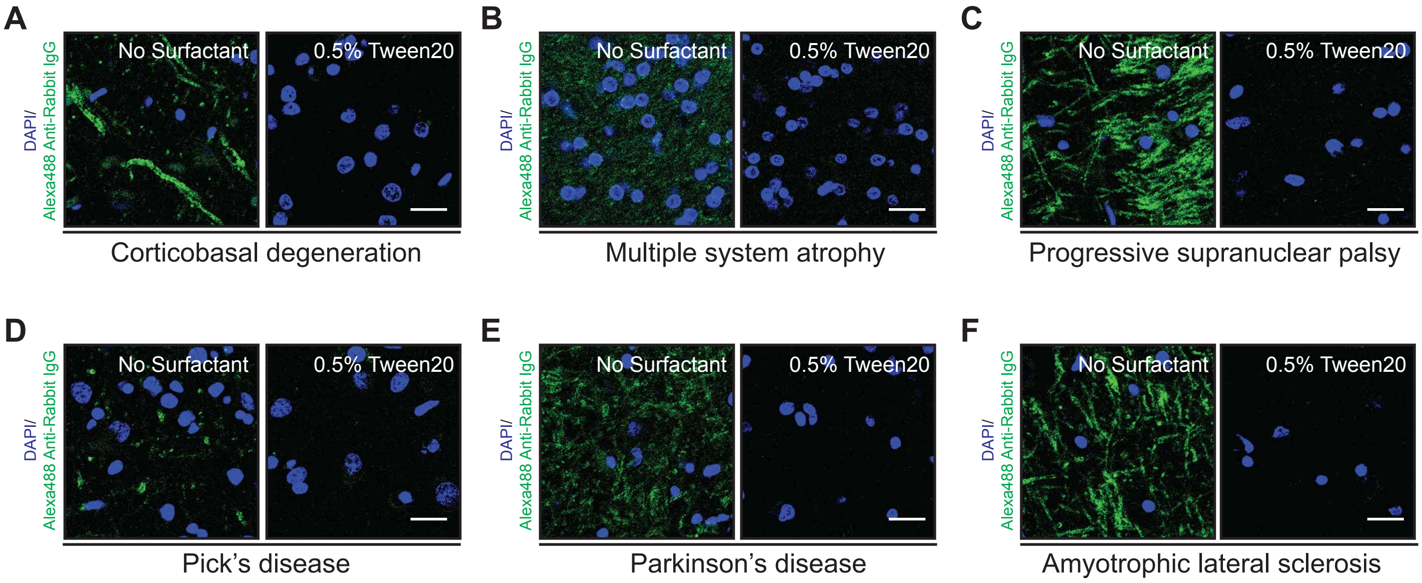
A
No Surfactant
0.5% Tween20
DAPI/
Alexa488 Anti-Rabbit IgG
Corticobasal degeneration
B
No Surfactant
0.5% Tween20
DAPI/
Alexa488 Anti-Rabbit IgG
Multiple system atrophy
C
No Surfactant
0.5% Tween20
DAPI/
Alexa488 Anti-Rabbit IgG
Progressive supranuclear palsy
D
No Surfactant
0.5% Tween20
DAPI/
Alexa488 Anti-Rabbit IgG
Pick's disease
E
No Surfactant
0.5% Tween20
DAPI/
Alexa488 Anti-Rabbit IgG
Parkinson's disease
F
No Surfactant
0.5% Tween20
DAPI/
Alexa488 Anti-Rabbit IgG
Amyotrophic lateral sclerosis

Supplementary Fig. S3

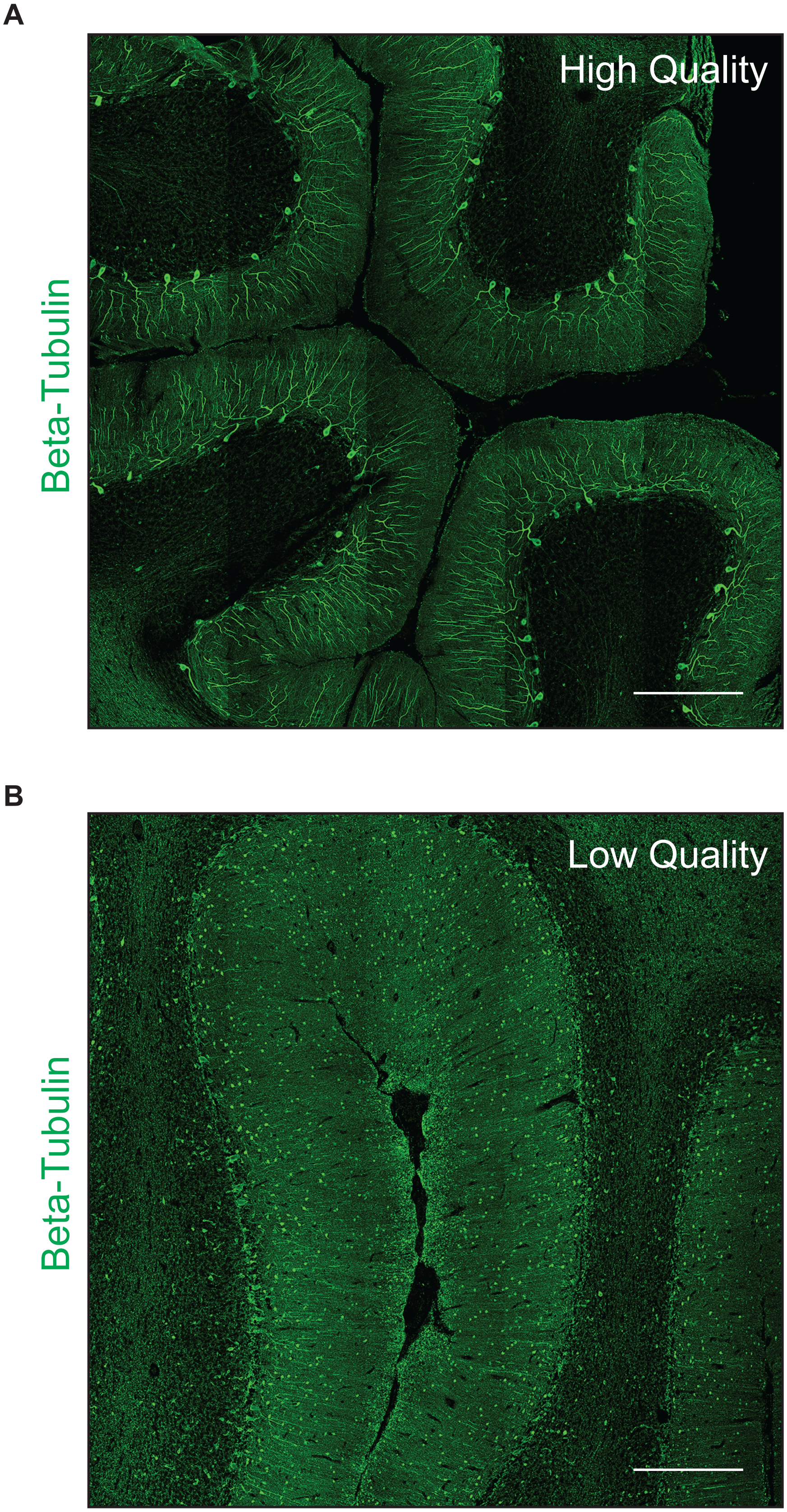

Supplementary Fig. S4

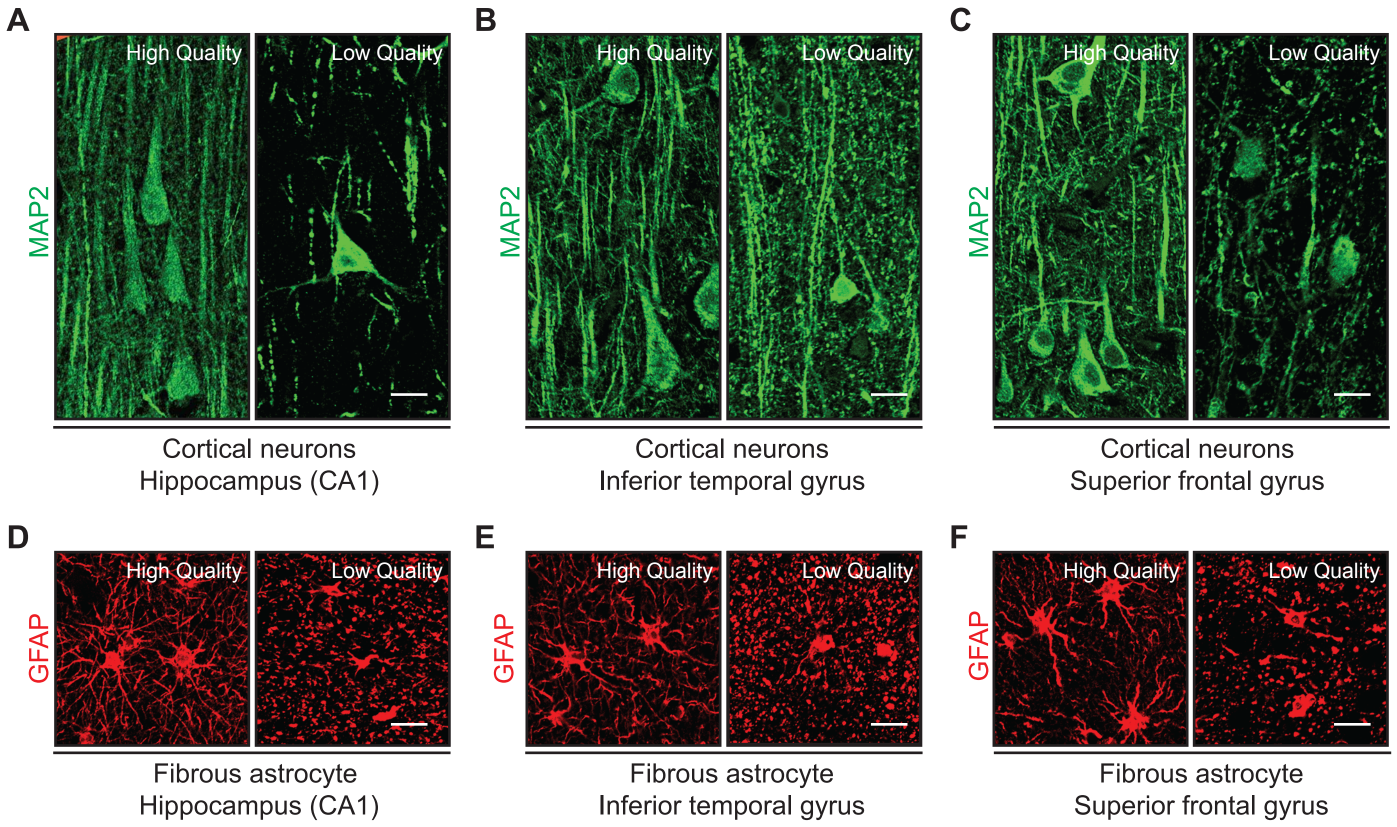

Supplementary Fig. S5

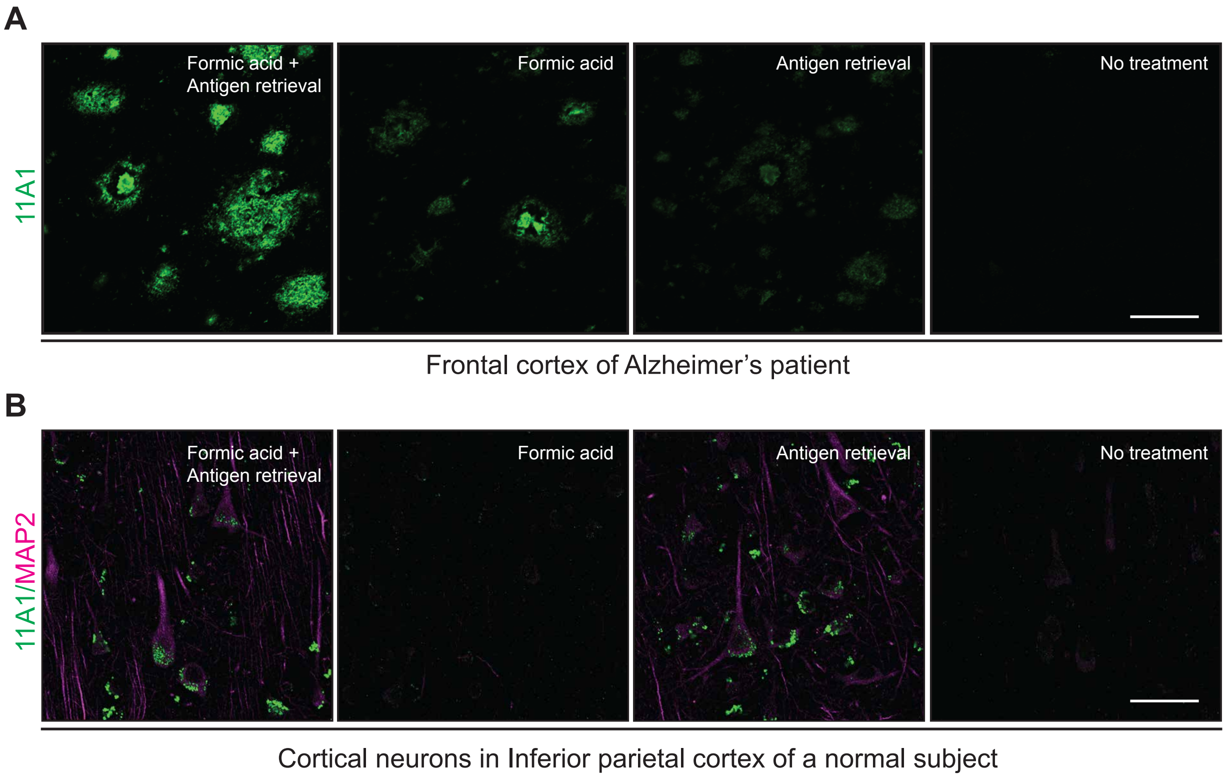

Supplementary Fig. S6

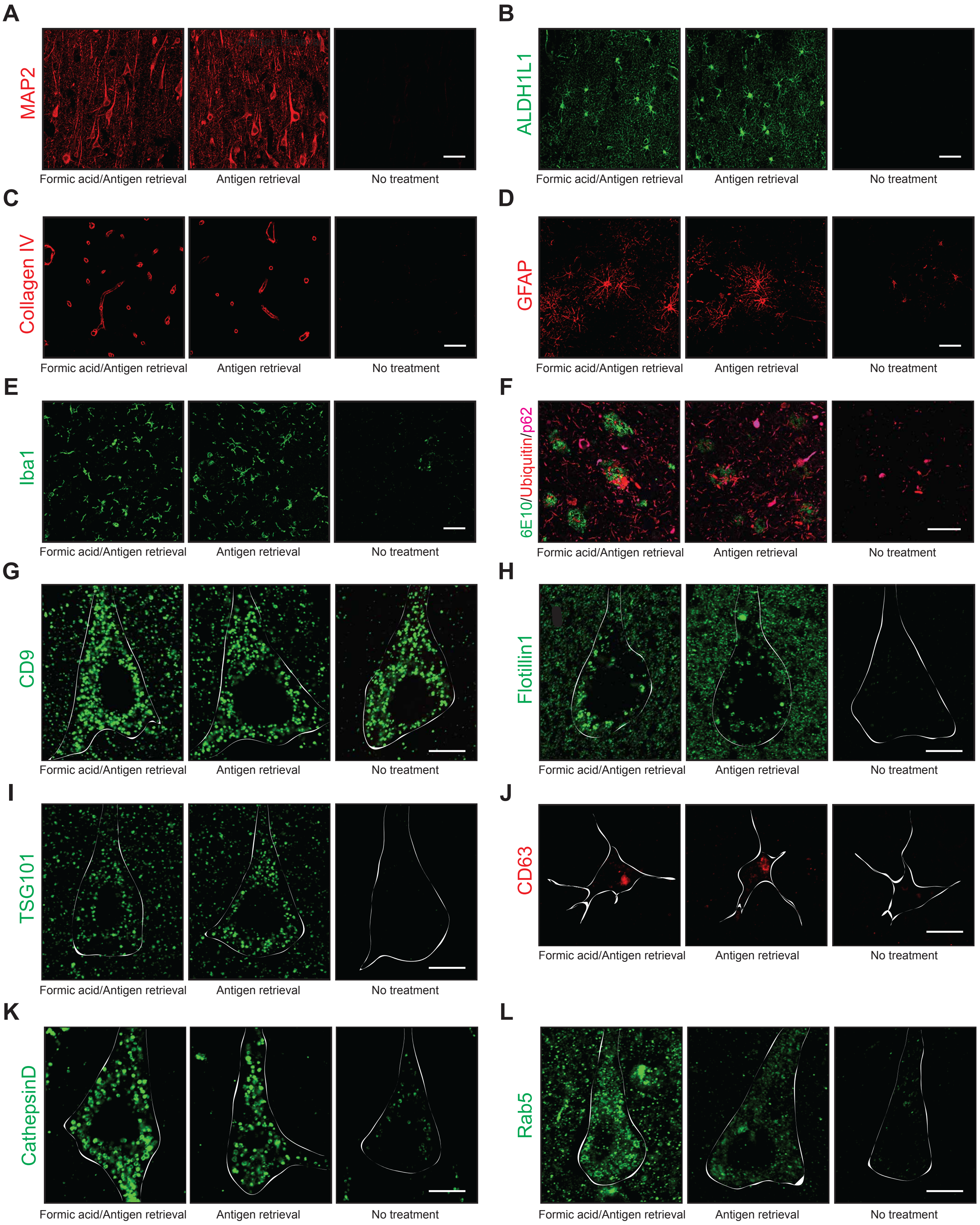

Supplementary Fig. S7

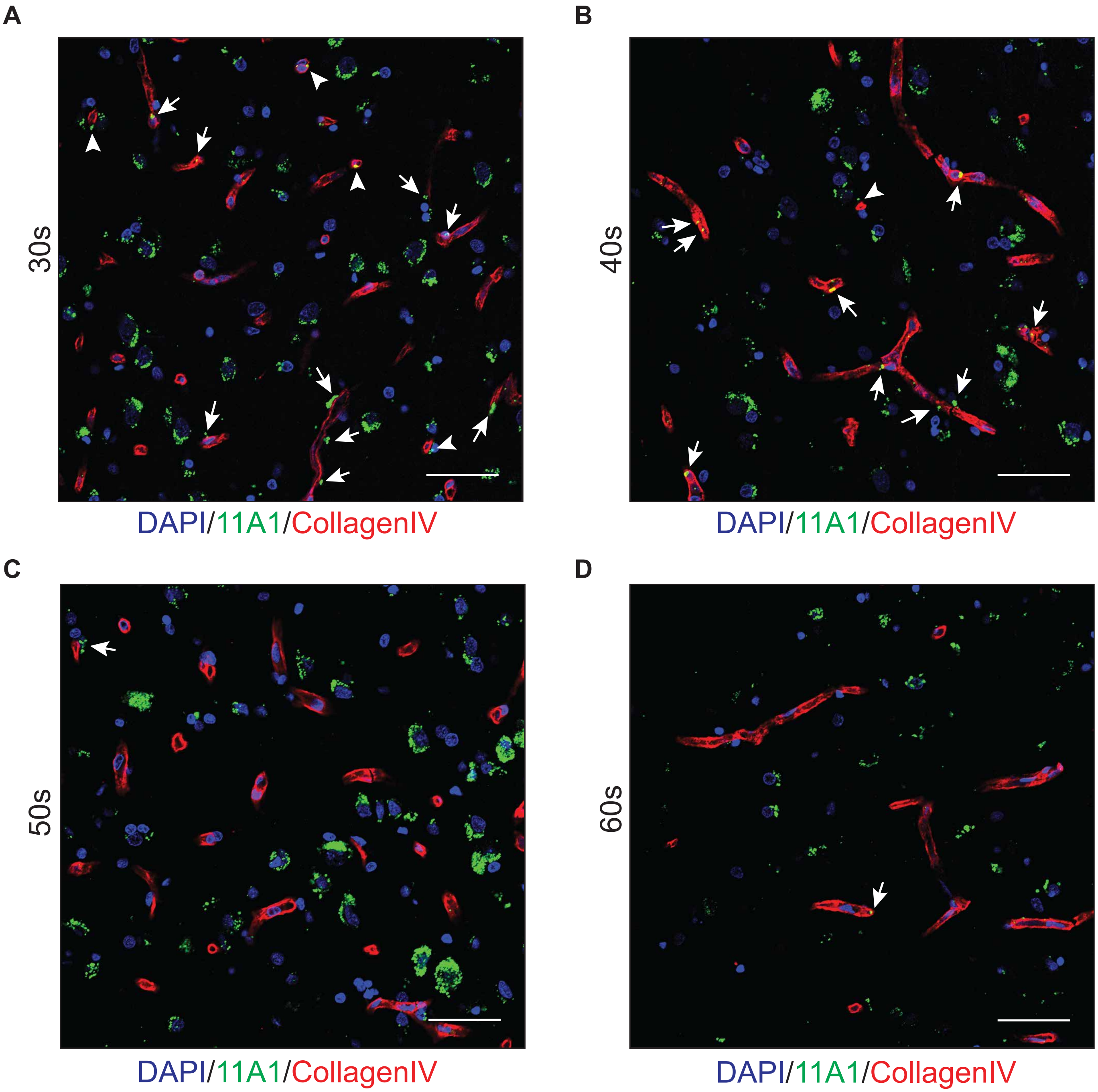

Supplementary Fig. S8

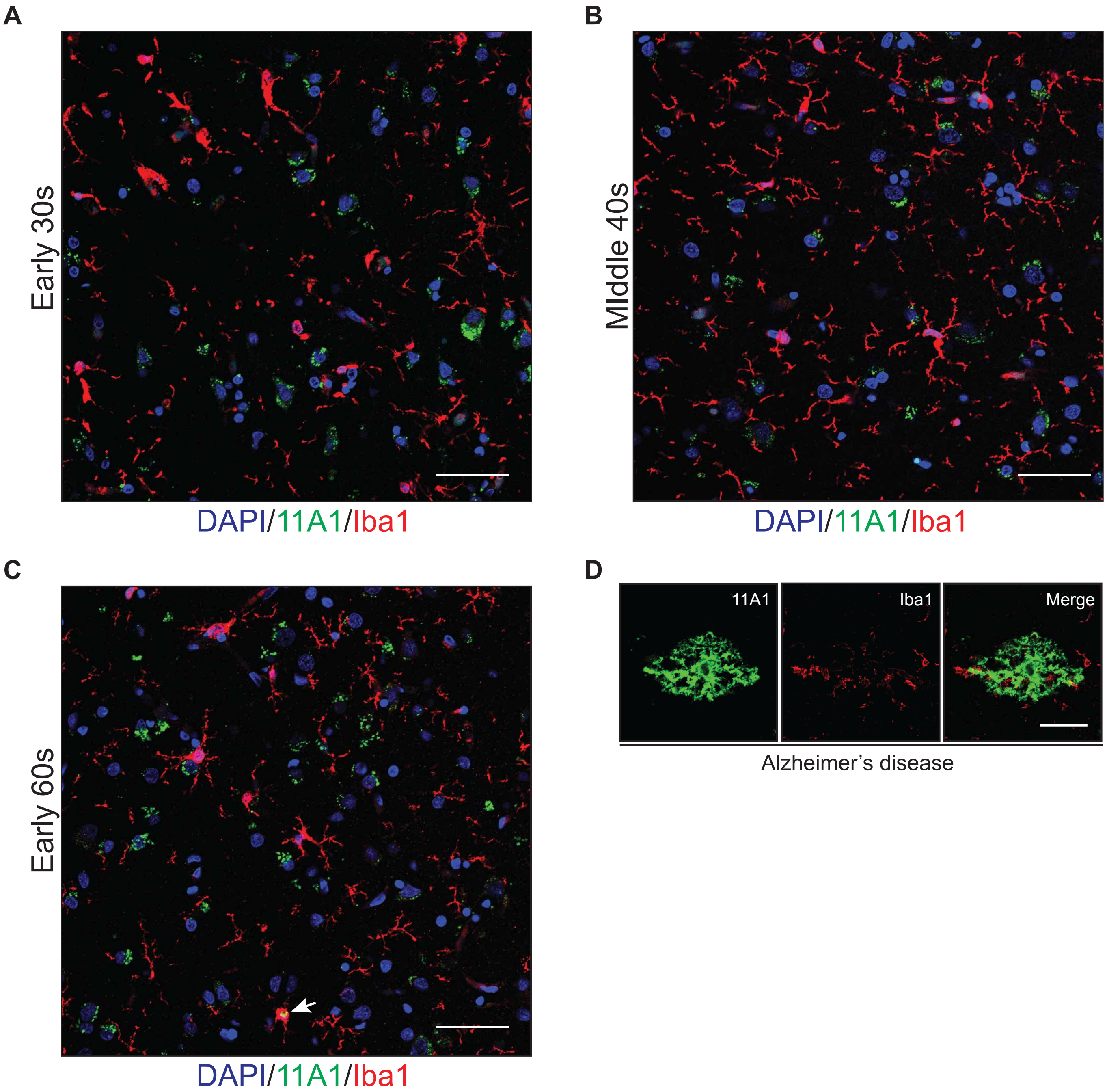

Supplementary Fig. S9

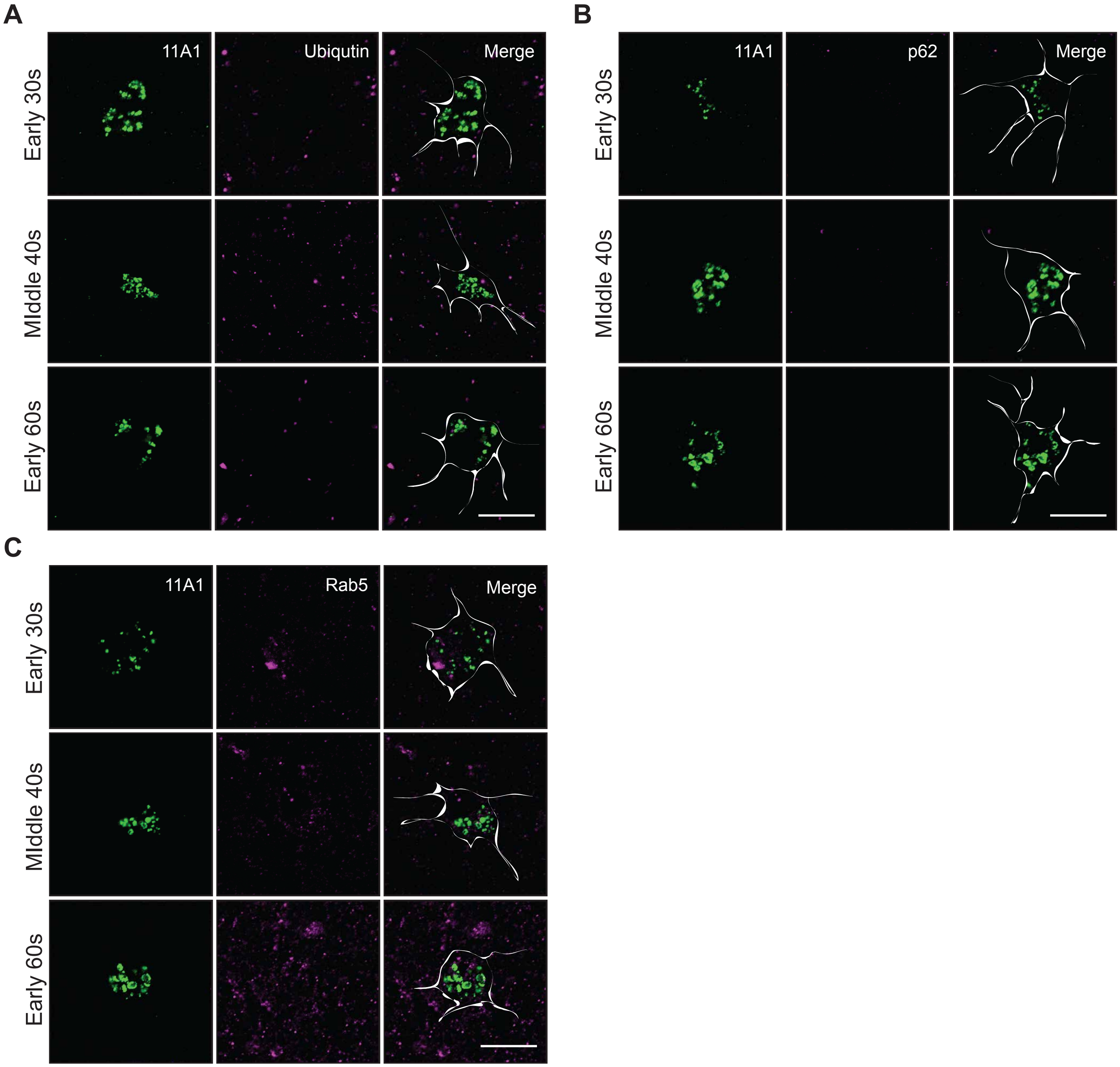

Supplementary Fig. S10

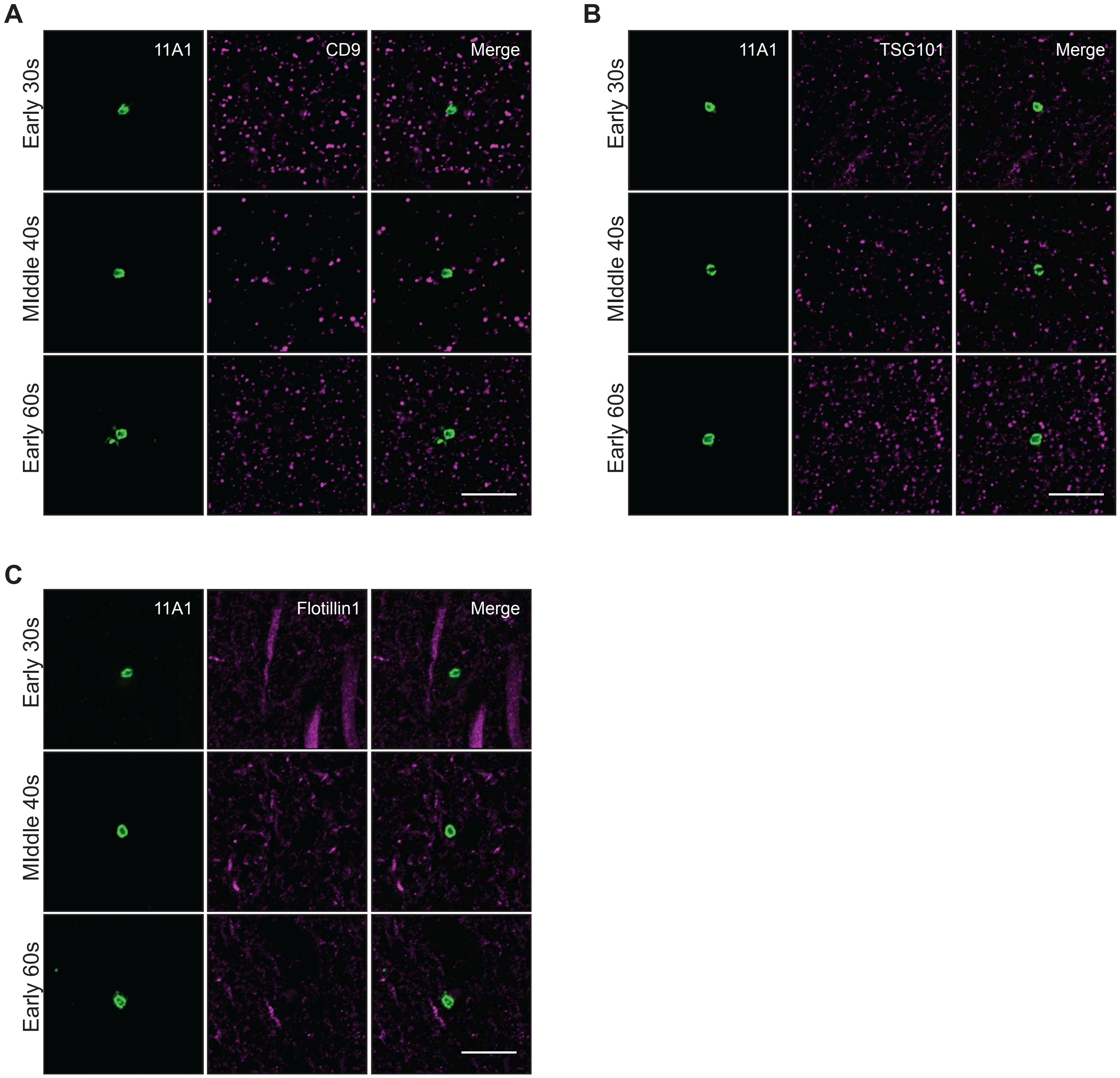

Supplementary Fig. S11

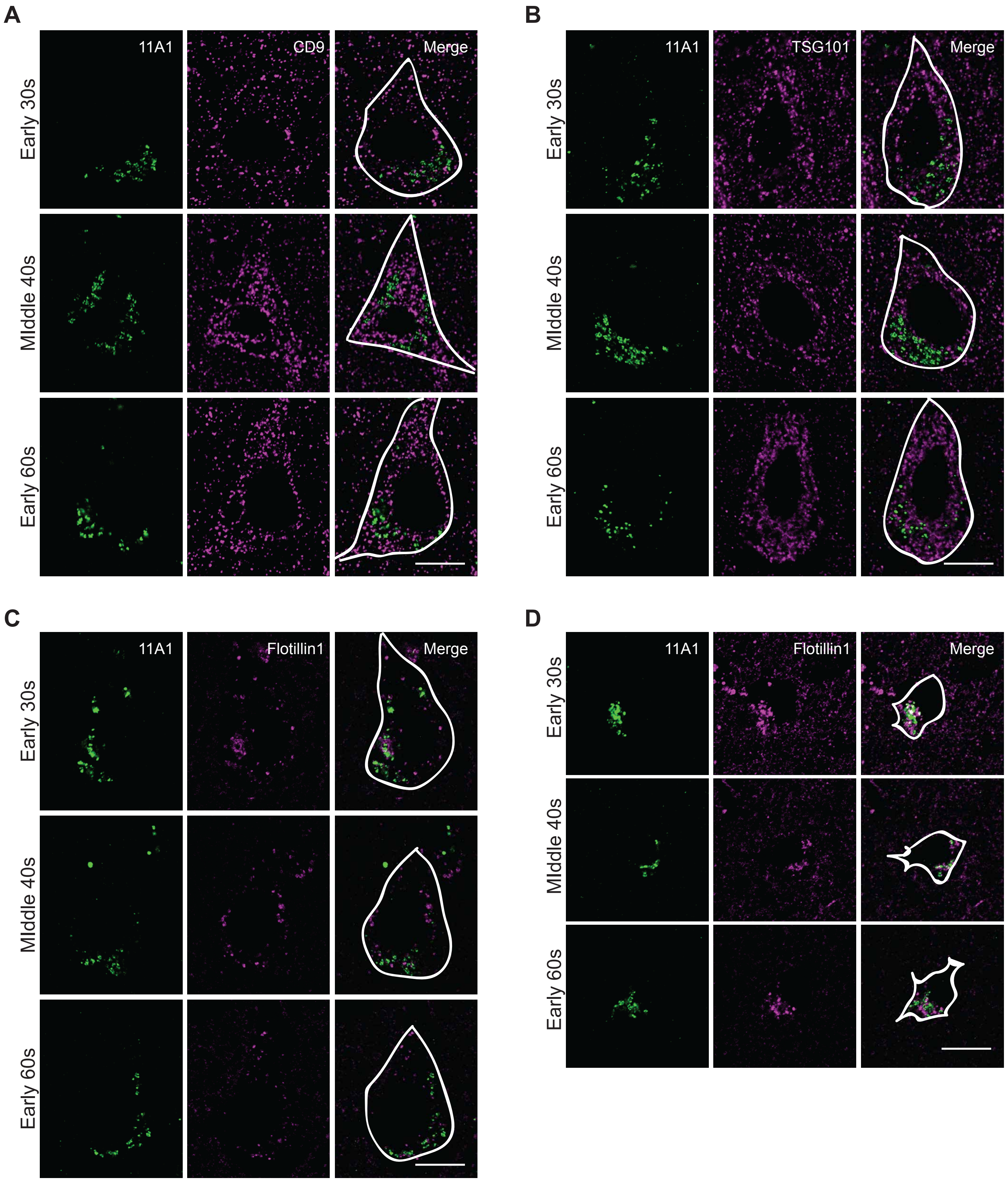

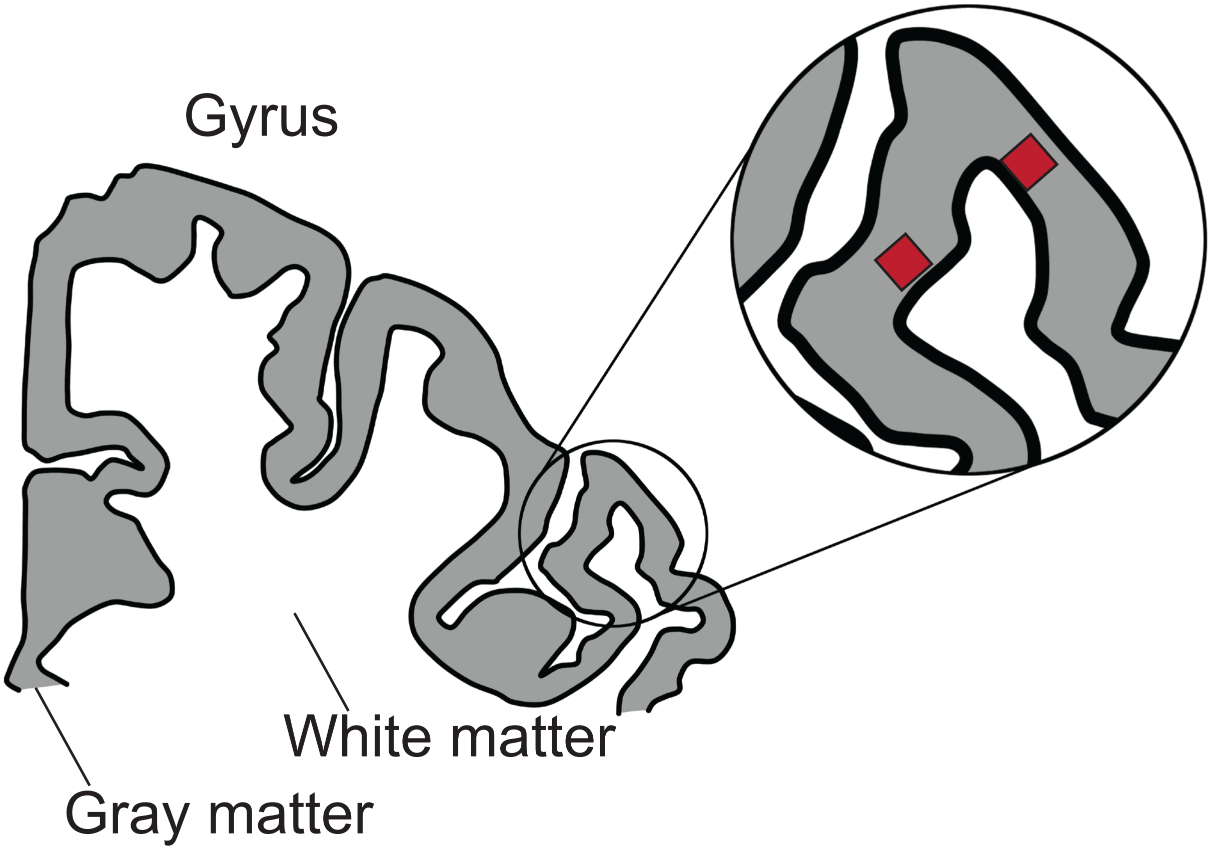
Gyrus
White matter
Gray matter

Supplementary Figure Legends

Supplementary Fig. S1. Quenching of lipofuscin autofluorescence signal with TrueBlack treatment in human brain neurons (A) CA1 hippocampus, **(B)** superior frontal gyrus, and **(C)** middle temporal gyrus. Confocal images of these neurons immunostained with MAP2 antibody are presented on the far left panels. The two central panels illustrate the autofluorescence of lipofuscin in the red and far-red channels. The far right panels show the merged images. The top panels of each image illustrate the results in the absence of TrueBlack treatment. The lower panel shows complete quenching of the lipofuscin signal with TrueBlack treatment. *All scale bars* 5µm.

Supplementary Fig. S2. Quenching of nonspecific binding of anti-rabbit IgG secondary antibody in human brain paraffin sections with 0.5% Tween20. Brain tissue sections from various neurodegenerative diseases show variable degrees of non-specific binding of anti-rabbit IgG as presented in the left panel of each image. This non-specific binding can be prevented by 0.5% Tween20 in the secondary antibody incubation buffer. **(A)** Corticobasal degeneration (inferior parietal lobule), **(B)** Multiple system atrophy (basal forebrain), **(C)** Progressive supranuclear palsy (Substantia nigra), **(D)** Pick's disease (frontal cortex), **(E)** Parkinson's disease (substantia nigra), and **(F)** Amyotrophic lateral sclerosis (Motor cortex). *All scale bars* 20µm.

Supplementary Fig. S3. Large field of Purkinje cells of the human cerebellum immunostained with Beta-tubulin antibody. (A) The high quality tissue sample shows structural integrity of the cell bodies, dendrites and axonal bundles in a mosaic image of a 5x5 panel captured with a 10x objective lens. **(B)** The low quality tissue sample shows loss of structure and attenuation of immunoreactivity in the cell bodies and dendrites. *All scale bars* 500µm.

Supplementary Fig. S4. Testing tissue quality and integrity of cortical neurons and astrocytes in high and low quality tissue samples defined by the structural integrity of Purkinje cells. (A-C) Images are of neurons immunostained with MAP2 antibody. The left panels show well preserved neuronal cell bodies and dendrites; whereas the right panels demonstrate fragmentation of dendrites and deformed cell bodies. **(D-F)** Images show white matter astrocytes immunostained with GFAP antibody in three brain regions. The left panels show intact astrocytic cell bodies and processes that are disintegrating in the right side panels. *All scale bars* 20 µm.

Supplementary Fig. S5. Effects of formic acid treatment and antigen retrieval on tissue staining. (A) Comparison of 11A1 antibody staining pattern in various tissue treatment conditions. Frontal cortex sections of an Alzheimer's disease patient were treated with formic acid/EDTA antigen retrieval (left panel), formic acid (center left panel), EDTA antigen retrieval (center right panel), or no treatment (right panel) prior to incubation with 11A1 antibody. 11A1 immunoreactivity against senile plaques was highest in formic acid/antigen retrieval treatment, less intense with formic acid alone, barely detectable with antigen retrieval alone, and absent with no treatment. **(B)** Inferior parietal cortex sections of a normal subject were treated with formic acid/EDTA antigen retrieval (left panel), formic acid (center left panel), antigen retrieval (center right panel), or no treatment (right panel) prior to incubation with 11A1 and MAP2 antibodies. Immunoreactivity of 11A1 and MAP2 antibodies were comparable in formic acid/EDTA antigen retrieval and EDTA antigen retrieval treatment. Formic acid treatment and no treatment showed no immunoreactivity. *All scale bars* 50µm.

Supplementary Fig. S6. Comparison of the staining pattern of all antibodies used in this study in various tissue treatment conditions. Inferior parietal cortex sections of a normal subject were treated with formic acid/EDTA antigen retrieval (left panel), EDTA antigen retrieval (middle panel), or no treatment (right panel) prior to incubation with the antibodies examined in this study. The immunoreactivity of all antibodies, except CD9, was enhanced by EDTA antigen retrieval treatment. Immunoreactivity of all antibodies was comparable between formic acid/EDTA antigen retrieval and EDTA antigen retrieval treatment. The white contours in the merged images indicates the cell shape identified by MAP2 (S7G, S7H, S7I, S7K, S7L) and GFAP (S7J). **(A)** MAP2, (**B)** ALDH1L1, **(C)** Collagen IV, **(D)** GFAP, **(E)** Iba1, **(F)** 6E10/Ubiquitin/p62, (**G)** CD9, **(H)** Flotillin1, **(I)** TSG101, **(J)** CD63, **(K)** Cathepsin D, **(L)** Rab5. *Scale bars* 50µm. (**A**-**F)**; 5µm (**G**- **L**).

Supplementary Fig. S7. 11A1 immunoreactivity in pericapillary spaces in various age groups. Tissue from inferior parietal cortex was co-stained with 11A1 (green) and Collagen IV (red) followed with DAPI staining: **(A)** 30s, **(B)** 40s, **(C)** 50s, and **(D)** 60s. Multiple 11A1 immunoreactive particles were noted in pericapillary spaces of subjects in their 30s and 40s, indicated with white arrows. The 11A1 immunoreactive particles in pericapillary spaces appear more abundant in 30- and 40-year-old subjects than in 50- and 60-year-old subjects. This reduction in 11A1 immunoreactive particles is noted both in cross-sectional (arrow heads) and longitudinal (arrows) capillary profiles. *All scale bars* 50µm.

Supplementary Fig. S8. Lack of 11A1 immunoreactivity in microglia. (A) Microglia recruitment to an Aβ plaque. Postmortem inferior parietal cortex of an Alzheimer's disease patient was co-stained with 11A1 and Iba1 antibodies. 11A1 (left panel), Iba1 (middle panel) and merged image (right panel). The merged image shows microglia infiltrating the Aβ plaque. **(B-D)** colocalization analysis of 11A1 immunoreactivity and microglia. Tissue from inferior parietal cortex was co-stained with 11A1 (green) and Iba1 (red) followed with DAPI staining: **B** early 30s, **C** middle 40s, and **D** early 60s. A single instance of colocalization is noted in the early 60s, indicated with a white arrow**,** but none in images of early 30s, or middle 40s. *All scale bars* 50µm.

Supplementary Fig. S9. Colocalization analysis of 11A1 immunoreactive particles and degradation markers in protoplasmic astrocytes. In all images, the rows correspond to three age groups: early 30s, middle 40s, and early 60s. Left panels show the 11A1 signal; middle panels correspond to the markers: **(A)** ubiquitin, **(B)** p62, **(C)** Rab5; and right panels show merged images. The white line in the merged images indicates the cell shape identified by GFAP signal. No obvious colocalization was noted. *All scale bars* 5µm.

Supplementary Fig. S10. Lack of colocalization of extracellular 11A1 immunoreactivity with various vesicle markers. Tissue from the inferior parietal cortex layer V was immunostained with 11A1 antibody (green) and three vesicle markers (magenta). **(A)** 11A1 & CD9, **(B)** 11A1 & TSG101**, (C)** 11A1 & Flotillin1. In all images, the rows correspond to three age groups: early 30s, middle 40s, and early 60s. 11A1 immunoreactive particles in neuropil were identified as described in the result section. No obvious colocalization was noted. *All scale bars* 5µm.

Supplementary Fig. S11. Colocalization analysis of the intracellular 11A1 immunoreactivity with various vesicle markers in neurons (A-C) and astrocytes (D). Tissue from the inferior parietal cortex layer V was immunostained with 11A1 antibody (green) and three vesicle markers (magenta). MAP2 or GFAP antibody was used as cell-type marker for neurons and astrocytes, respectively. These immunostains are not shown, but were used to delineate cell

shapes (in white). CD9 immunoreactivity and TSG101 immunoreactivity were found in MAP2 positive pyramidal neurons, not in GFAP positive astrocytes. Flotillin1 immunoreactivity was found in both MAP2 positive pyramidal neurons and GFAP positive astrocytes. **(A)** 11A1 & CD9 (MAP2), **(B)** 11A1 & TSG101 (MAP2)**, (C)** 11A1 & Flotillin1 (MAP2), and **(D)** 11A1 & Flotillin1 (GFAP). In all images, the rows correspond to three age groups: early 30s, middle 40s, and early 60s**.** No obvious colocalization was noted for any marker, except for Flotillin 1 in astrocytes in the early 30s in D**.** *All scale bars* 5µm.

Supplementary Fig. S12. Depiction of gyri of the inferior parietal cortex and the regions of interest (ROI) for analyses indicated by colored squares. 3x3 panel montage images covering 0.910 mm^2 were captured with x20 objective lens from layer V. Red square indicates ROI, however, size of square does not represent actual size.

Supplementary Table S1. Demography of neurodegenerative disease case brains which were used in this study

Sample ID	Pathological Diagnosis	Clinical History	Sex	Age	Figure
ND1	Alzheimer's disease	Dementia	F	80	Fig. 1B
ND2	Corticobasal degeneration	Language impairment, Parkinsonian syndrome	F	78	Supplementary Fig. S2A
ND3	Multiple system atrophy (MSA)	Gait disorder, Parkinsonian syndrome	F	90	Supplementary Fig. S2B
ND4	Progressive supranuclear palsy (PSP)	Parkinsonian syndrome, Dementia	M	79	Supplementary Fig. S2C
ND5	Pick's disease	Frontotemporal dementia	F	72	Supplementary Fig. S2D
ND6	Parkinson's disease	Parkinson's disease, Dementia	M	80	Supplementary Fig. S2E
ND7	Amyotrophic lateral sclerosis (ALS)	Motor neuron disease, Dementia, Aphasia	M	74	Supplementary Fig. S2F
ND8	Alzheimer's disease	Frontotemporal dementia	F	61	Fig. 2B, Supplementary Fig. S5A & S6F

Supplementary Table S2: Akaike Information Criterion (AIC) values

AIC	Fig 3D	Fig 3E	Fig 4D	Fig 4E	Fig 5D	Fig 5E	Fig 6E
Intercept only model	244.4	301.5	215.2	319.5	254.8	285.4	239.1
Linear age model	234.4	303.0	217.2	321.2	251.3	281.3	217.9
Piecewise model	232.9	305.0	216.3	320.6	252.2	282.8	216.8

Red indicates the best fitted model.

Supplementary Table S3: List of primary antibodies used in this study

Antibody	Company	Catalog #	Host	Dilution	Target
MAP2	Abcam	ab92434	Chicken	1/1000	Neuron
Class 3 B-tubulin	Abcam	ab18207	Rabbit	1/1000	Neuronal beta tubulin
GFAP	DAKO	Z-0334	Rabbit	1/200	Astrocyte
GFAP	Abcam	Ab4674	Chicken	1/1000	Astrocyte
ALDH1L1	Abcam	ab177463	Rabbit	1/100	Astrocyte
Iba1	WAKO	019-19741	Rabbit	1/200	Microglia
Collagen IV	Abcam	Ab6586	Rabbit	1/500	Collagen in perivascular space
11A1	IBL	10379	Mouse	1/50	Amyloid β oligomer
6E10	Biolegend	SIG-39320	Mouse	1/500	Amyloid β
4G8	Biolegend	800701	Mouse	1/500	Amyloid β
Rab5	Abcam	ab109534	Rabbit	1/200	Early Endosome
LC3 A/B	Abcam	ab128025	Rabbit	1/200	Autophagosome
p62	Progen	GP62-C	Guinea pig	1/200	Autophagosome
Cathepsin D	Abcam	ab75852	Rabbit	1/200	Lysosome
CD63	Abcam	Ab134045	Rabbit	1/500	Tetraspanin family protein
CD9	Abcam	Ab92726	Rabbit	1/500	Tetraspanin family protein
Flotillin1	Abcam	Ab133497	Rabbit	1/100	Caveole-associated Integral membrane protein
TSG101	Abcam	Ab125011	Rabbit	1/500	Component of the ESCRT-1 complex

Supplemetary Table S4: List of secondary antibodies used in this study

Antibody	Company	Catalog #	Host	Dilution
Anti-Mouse IgG H&L (Alexa Fluor 488)	Abcam	ab150117	Goat	1/400
Anti-Mouse IgG H&L (Alexa Fluor 568)	Abcam	ab175701	Goat	1/400
Anti-Rabbit IgG H&L (Alexa Fluor 488)	Abcam	ab150081	Goat	1/400
Anti-Rabbit IgG H&L (Alexa Fluor 568)	Abcam	ab175696	Goat	1/400
Anti-Rabbit IgG H&L (Alexa Fluor 647)	Abcam	ab150083	Goat	1/400
Anti-Chicken IgY H&L (Alexa Fluor 488)	Abcam	ab150173	Goat	1/400
Anti-Chicken IgY H&L (Alexa Fluor 568)	Abcam	ab175711	Goat	1/400
Anti-Chicken IgY H&L (Alexa Fluor 647)	Abcam	ab150175	Goat	1/400
Anti-Guinea pig IgG H&L (Alexa Fluor 568)	Abcam	ab175714	Goat	1/400
Anti-Guinea pig IgG H&L (Alexa Fluor 647)	Abcam	ab150187	Goat	1/400

*All antiserum, except ab150187 and ab175714, were cross adsorbed using bovine, horse, human, mouse, pig, rabbit and rat immunosorbents to remove cross reactive antibodies. (Manufacturer's note)
